# High IGKC-Expressing Intratumoral Plasma Cells Predict Response to Immune Checkpoint Blockade

**DOI:** 10.3390/ijms23169124

**Published:** 2022-08-15

**Authors:** Juan Luis Onieva, Qingyang Xiao, Miguel-Ángel Berciano-Guerrero, Aurora Laborda-Illanes, Carlos de Andrea, Patricia Chaves, Pilar Piñeiro, Alicia Garrido-Aranda, Elena Gallego, Belén Sojo, Laura Gálvez, Rosario Chica-Parrado, Daniel Prieto, Elisabeth Pérez-Ruiz, Angela Farngren, María José Lozano, Martina Álvarez, Pedro Jiménez, Alfonso Sánchez-Muñoz, Javier Oliver, Manuel Cobo, Emilio Alba, Isabel Barragán

**Affiliations:** 1 Medical Oncology Intercenter Unit, Instituto de Investigación Biomédica de Málaga y Plataforma en Nanomedicina–IBIMA Plataforma Bionand, Regional and Virgen de la Victoria University Hospitals, 29010 Malaga, Spain; 2 Cancer Molecular Biology Laboratory (LBMC), Translational Research in Cancer Immunotherapy Group, Health and Medical Research Centre (CIMES), University of Malaga (UMA), Marques de Beccaria 3, 29010 Malaga, Spain; 3 Instituto de Investigación Biomédica de Málaga y Plataforma en Nanomedicina–IBIMA Plataforma Bionand, 29010 Malaga, Spain; 4 Facultad de Medicina, Campus de Teatinos s/n, Universidad de Málaga, 29071 Malaga, Spain; 5 Section of Pharmacogenetics, Department of Physiology and Pharmacology, Karolinska Institutet, 17177 Stockholm, Sweden; 6 Group of Pharmacoepigenetics, Department of Physiology and Pharmacology, Karolinska Institutet, 17177 Stockholm, Sweden; 7 Department of Anatomy, Physiology and Pathology, University of Navarra, 31008 Pamplona, Spain; 8 Department of Anatomy and Pathology, University of Navarra, 31008 Pamplona, Spain; 9 Department of Pathology, Clinical University Hospital, Campus de Teatinos, 29010 Malaga, Spain; 10 Department of Pathology, University of Malaga (UMA), 29010 Malaga, Spain

**Keywords:** immunotherapy, biomarkers, melanoma

## Abstract

Resistance to Immune Checkpoint Blockade (ICB) constitutes the current limiting factor for the optimal implementation of this novel therapy, which otherwise demonstrates durable responses with acceptable toxicity scores. This limitation is exacerbated by a lack of robust biomarkers. In this study, we have dissected the basal TME composition at the gene expression and cellular levels that predict response to Nivolumab and prognosis. BCR, TCR and HLA profiling were employed for further characterization of the molecular variables associated with response. The findings were validated using a single-cell RNA-seq data of metastatic melanoma patients treated with ICB, and by multispectral immunofluorescence. Finally, machine learning was employed to construct a prediction algorithm that was validated across eight metastatic melanoma cohorts treated with ICB. Using this strategy, we have unmasked a major role played by basal intratumoral Plasma cells expressing high levels of IGKC in efficacy. IGKC, differentially expressed in good responders, was also identified within the Top response-related BCR clonotypes, together with IGK variants. These results were validated at gene, cellular and protein levels; CD138+ Plasma-like and Plasma cells were more abundant in good responders and correlated with the same RNA-seq-defined fraction. Finally, we generated a 15-gene prediction model that outperformed the current reference score in eight ICB-treated metastatic melanoma cohorts. The evidenced major contribution of basal intratumoral IGKC and Plasma cells in good response and outcome in ICB in metastatic melanoma is a groundbreaking finding in the field beyond the role of T lymphocytes.

## 1. Introduction

Checkpoint inhibitor therapy constitutes a promising cancer treatment strategy that targets the immune checkpoints to reactivate silenced T-cell cytotoxicity. In recent pivotal trials, Immune Checkpoint Blockade (ICB) demonstrated durable responses and acceptable toxicity, resulting in the regulatory approval of eight checkpoint inhibitors for 15 cancer indications. Indeed, in metastatic melanoma, anti-PD1 is the standard choice for first-line treatment (Checkmate-066, Keynote006). However, up to ~85% of patients present innate or acquired resistance to ICB, limiting its clinical utility [1,2,3]. In addition to the subsequent impact on patient survival of the lack of efficacy in most patients, the identification of resistance biomarkers for patient selection is critical given the escalating costs of this type of treatment, which currently ranges from USD 50,000 to 100,000/quality-adjusted life year [4]. So far, only PD-L1, quantified by immunohistochemistry, stands as an FDA/EMA approved biomarker of response to ICB for the selection of candidates to be treated with monotherapy anti-PD1 in NSCLC [5] and recurrent or metastatic squamous cell carcinoma of the head and neck [6], but not in melanoma. Moreover, its utility in selecting patients for therapy is hampered by the unclear definition of PD-L1 positivity and at least some potential for therapeutic response, regardless of tumor PD-L1 status [7,8]. Indeed, even though four IHC assays have been approved by the FDA, protein PD-L1 expression fails to accurately predict the response to ICB in some cases [9]. In part, this is due to the observed intra-tumor heterogeneity of PD-L1 expression [10]. The exploration of other molecular and cellular biomarkers of response is still limited, with relatively modest and retrospective cohorts [11,12,13,14,15,16,17,18,19,20,21,22,23,24,25,26,27,28]. Thus, identification of novel, more predictive biomarkers that could identify patients who would benefit from ICB constitutes one of the most important areas of immunotherapy (IT) research [29]. In this work, we used coding and non-coding transcriptome analysis in bulk tumor samples from melanoma patients treated with the anti-PD1 agent Nivolumab to identify a signature of 140 genes predictive of response, of which a number (55 for PFS and 59 for OS) were also prognostic. Interestingly, the signature unmasked a pattern of high B lymphocyte activity under response. This was also manifested in a significant enrichment in BCR isotopes and abundance. The integration with single-cell transcriptome datasets from melanoma patients treated with ICB (anti-PD1, anti-CTLA4 and anti-CTLA4 plus anti-PD1) led to the refinement of a specific B lineage-related subtype associated with response: Plasma cells. Importantly, it also served for the biological validation of the B-cell-related response signature at the level of individual genes and associated cell populations and lymphoid structures. Our results will pave the way for de-complexing the interactions among the tumor and the different immune cells implicated in the reactivation of the anti-tumor response associated to ICB.

## 2. Results

### 2.1. Clinicopathological Variables Associated with Response and Prognosis

The univariate analysis of clinical variables only resulted in the association of lung metastasis to response to Nivolumab when analyzing the cutaneous melanoma cohort. When the cohort was expanded to include both cutaneous and non-cutaneous melanoma, the Likelihood ratio test *p*-value indicated an even higher significance. Additionally, in the expanded cohort, stage at diagnosis and IT toxicity were associated significantly with response (Table S1).

Regarding prognosis, cutaneous melanoma patients with lung metastasis have greater progression-free survival (PFS) with a median PFS of 354 days (95% CI, 180—NR), compared with patients without lung metastasis, who have a median PFS of 55 days (95% CI, 16—NR). Previously, there was no correlation between increased survival and patients with melanoma and lung metastases treated with IT. This relation will have to be confirmed in a wider group of patients. In terms of overall survival (OS), lung metastasis is also associated with a better outcome. The median OS of patients with lung metastasis is 869 days (95% CI, 321—NR), while patients without lung metastasis have a median OS of 39 days (95% CI, 28—NR). In contrast, lymph node metastasis is associated with worse survival: median OS of patients with lymph node metastasis is 88.5 days (95% CI, 35—NR), and with no metastasis, the median OS is 490 days (95% CI, 490—NR) (Table S2). In addition to the above variables, there are two other parameters that present different survival distribution among the patients: stage at diagnosis and IT toxicity status. While Stage IV is associated with the lowest OS (median OS of 46.5 days (95% CI, 26—NR)), suffering from Nivolumab-induced toxicity is related to increased OS, with a median OS of 563 (95% CI, 321—NR).

### 2.2. B-Cell Transcriptomic Signature of the Response to PD1 Blockade

**Figure 1:**
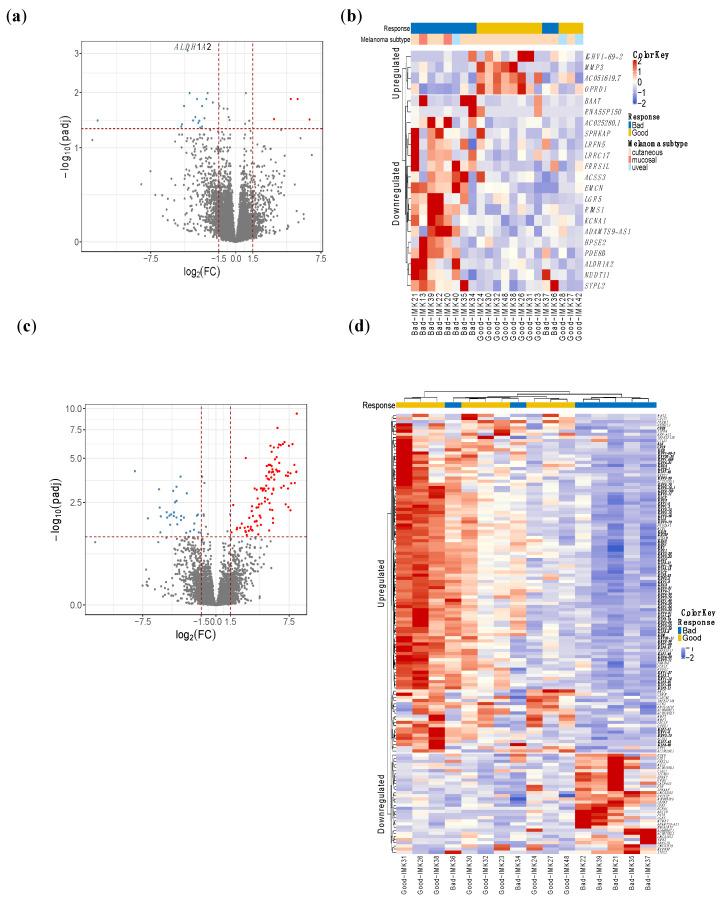
**DE genes in responders of all types of melanomas and cutaneous melanomas.** (**a**) and (**c**) volcano plot. The red dots depict genes that are over-expressed (*x*-axis positive section), or under-expressed (*y*-axis negative section) in responders to Nivolumab. (**b**,**d**) heatmap showing the hierarchical clustering of good and bad responders based on the expression of the DE genes. Analysis of all types of melanomas identified 22 DE genes (**a**,**b**), whereas in cutaneous melanoma, we obtained 140 DE genes (**c**,**d**). (**d**) Highlighted genes in bold are related to immunoglobulins and B-cell activity.

Given that our cohort comprised three types of melanoma that are molecularly distinct, we employed two different approaches to identify the response-relevant genes. Initially, the joint analysis with all subtypes yielded 22 DE genes in good responders, given the heterogeneous nature of the cohort in terms of melanoma types. The gene *Aldehyde Dehydrogenase 1 Family Member A2* (*ALDH1A2*) was the most differentially expressed in this cohort (Figure 1a,b, Table S3).

On the other hand, the interrogation of the differential expression in the subgroup of 16 cutaneous melanoma patients yielded 140 genes with a significant difference in expression (Table S3, Figure 1c,d). This indicates a greater difference in magnitude between the compared groups of patients, probably because of the greater baseline homogeneity of the patients with cutaneous melanoma.

Out of the 140 DE genes, 73 code for different domains of immunoglobulins as well as B-cell receptors such as CD19 and CD22, showing important upregulation of the B-cell activity in the immune infiltrate of the tumor.

For those 140 DE genes in the cutaneous metastatic melanoma patients, we obtained 22 biological processes (BP) involved, 9 molecular functions (MF) and 15 cellular components (CC) that are significantly enriched by Gene Ontology (GO) analysis, and are shown in Table S4. Enrichment analysis by biological processes demonstrated a large response–relation difference in biological processes related to B lymphocytes, including phagocytosis, complement activation and activation of B cells, as well as the production of immunoglobulins between good responders and bad responders to PD blockade. Similarly, immunoglobulin receptor binding and the monomeric immunoglobulin A complex were identified in enrichment analyses by molecular function and cellular components.

These results indicate that the presence or function of B lymphocytes in the tumor microenvironment (TME) may be involved in the response to anti-PD1 immune checkpoint inhibition IT.

### 2.3. TNFRSF11B, IGLV6-57, IGHA1 and GRIA1 Are Technically Validated as Promising Markers of Response to Nivolumab

Our RNA-sequencing data were technically validated by RT-PCR in a selection of genes representative of the different patterns of expression in the RNA-seq results, expressed in Transcripts Per Kilobase Million (TPM) (*TNFRSF11B, IGLV6-57*, *IGHA1* and *GRIA1*). In concordance with the RNA-sequencing expression levels, the genes involved in the response mechanisms linked to the immune system, and in particular the B-cell signature, (*TNFRSF11B*, *IGLV6-57*, *IGHA1*), were highly expressed in good responders, while the genes related to tumor-associated mechanisms of response (*GRIA1*), were highly expressed in bad responders (Figure S1a).

To statistically evaluate the concordance in gene expression intensities between RNA-sequencing from the Discovery cohort and qPCR from the technical validation experiment, the correlations between the qPCR ∆Ct values and the RNA-sequencing log-transformed TPM values were calculated using Pearson’s correlation coefficient (Figure S1b, Table S5).

The overall correlation had a coefficient of 0.78 (*p* < 0.001), with individual gene correlation coefficients ranging from 0.80 to 1, showing consistent concordance of the validation and discovery expression differences between good responders and bad responders.

### 2.4. A Fraction of the Transcriptomic Signature of Response Is Prognostic

**Figure 2:**
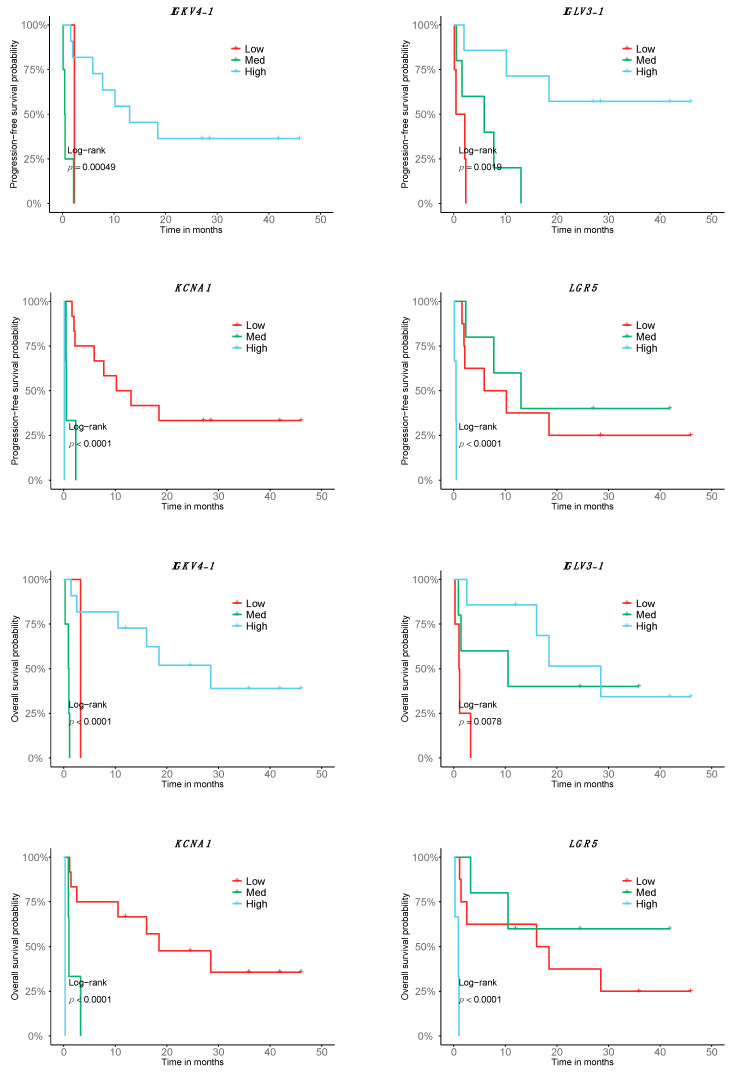
**Association of the transcriptomic signature of response to Nivolumab with prognosis.** Depiction of the top 8 genes with correlating expression with OS and PFS according to a stratification in low, high and medium expression (n = 16; 9 good responders, 7 bad responders).

In order to test the prognostic predictability of the transcriptome response signature, we tested it for its association with PFS and OS. Of the 140 DE genes in the good responders of the metastatic cutaneous melanoma cohort, 55 were significant in relation to patient PFS and 59 with respect to OS; that is, high, medium or low expression levels were correlated with longer or shorter survival. Interestingly, 35 of them are B-cell specific. While the expression of most B-cell-related genes is associated with better survival in both PFS and OS, there are some tumor-related genes in which the expression is associated with a decrease in survival, such as *LGR5* or *KCNA1* (Figure 2, Table S6). Among all the genes, 47 were significantly associated with both OS and PFS, whereas 12 genes were specific to OS and 8 genes were specific to PFS (Table S6).

### 2.5. TMB Is Not Associated with Response to Nivolumab in Cutaneous Metastatic Melanoma Patients

TMB, a biomarker for PD1 blockade in melanoma, has been reported to positively predict survival [20]. We therefore quantified and evaluated the TMB from our bulk RNA-seq data in the fraction of cutaneous metastatic melanomas. However, no statistical differences were found between the groups of good responders and bad responders (Figure S2a).

### 2.6. Decoding the Response-Relevant Mutational Signatures in Patients with Cutaneous Metastatic Melanoma

Given the established somatic mutation signatures of melanoma, we annotated the 16 cutaneous metastatic melanoma samples of the Discovery cohort with 11 Doublet Base Substitution (DBS) COSMIC (v3.2) mutational signatures with the aim of evaluating their putative association with the response to Nivolumab. A clear abundance of DBS1 (ultraviolet light exposure), DBS3 (polymerase epsilon exonuclease domain mutations), DBS7 (defective DNA mismatch repair) and DBS11 (unknown, possibly related to APOBEC mutagenesis) was identified among all patients irrespective of their response to Nivolumab. DBS2 (tobacco smoking and other mutagens; age of cancer diagnosis) and DBS4 (unknown; proposed etiology: age of cancer diagnosis) were more frequent among good responders (*p*-value < 0.05), whereas they were absent in some bad responders, including the severe bad responders IMK34 and IMK35. DBS6 (unknown) was also significantly increased in good responders; however, in contrast to the other two mutational signatures, it was also present in the rest of the samples (Figure S2b).

### 2.7. Identification of the Specific Response-Associated Stromal Cell Subtypes in Single Cell RNA-seq Data

The use of a single-cell RNA-seq cohort of metastatic melanoma patients treated with ICB [30,31] has allowed us to delineate specific cell populations through expression data.

**Figure 3:**
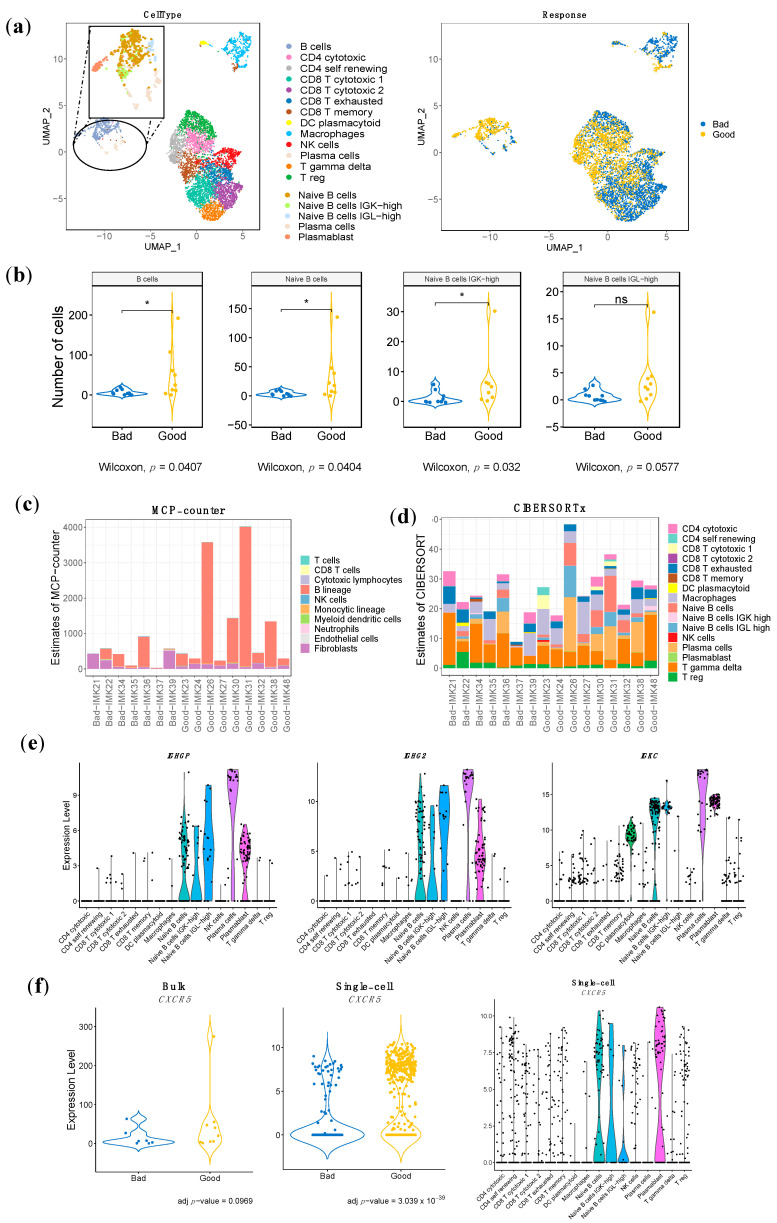
**Distribution and enrichment according to response of the stromal immune cells of the single-cell RNA-seq validation cohort, estimation of the population abundance by bulk RNA-seq transcriptomic deconvolution and bulk RNA-seq gene signature mapping to single cell RNA-seq.** (**a**) UMAP representation of the stromal cells, different types of immune cells. Each dot represents a single cell, and they are clustered and colored according to each cell type and according to the pattern of response. Increased resolution in B-cell lineage was performed to identify Naïve B cells, Naïve B cells IGK-high, Naïve B cells IGL-high, Plasma cells and Plasmablasts. (**b**) Comparison of the abundance among good and bad responders in B cells and the refined B-cell clusters. (**c**) and (**d**) Estimation of cell-population abundance using gene expression profile based on gene markers (**c**) (MCP-counter) and based on a custom signature matrix based on scRNA-seq analysis (**d**) (CIBERSORTx). (**e**) Violin plot of the gene expression in the scRNA-seq of three representative genes from our gene signature stratified by cell type validated in the scRNA-seq analysis. (**f**) Validation of the presence of the gene *CXCR5*, which is associated with tertiary lymphoid structures, in both bulk RNA-seq and scRNA-seq. *CXCR5* expression distribution over cell types in scRNA-seq. In (**b**) *p*-value < 0.05 is shown as *; ns refers to non-significant.

Top marker characterization has enabled us to distinguish between the following cell types: B cells, CD4 T cytotoxic, CD4 T self-renewing, CD8 T cytotoxic, CD8 T exhausted, CD8 T memory, DC plasmacytoid, Macrophages, NK cells, Plasma cells, T gamma delta cells and T-reg cells (Figure 3a). Additionally, we have managed to increase the resolution of the B-cell lineage to refine it into four different cell subtypes representing known functions and differentiation stages, as well as phenotypes that have not been characterized before and might be induced by the tumor: Naïve B cells, featured by *MS4A1*, *IRF8*, *BANK1*, *CD22*, and *TXNIP* expression; Naïve B cells Immunoglobulins Kappa Light chains (IGK)-high, with the following specific expression markers: *IGKV1-39*, *IGKV1-12*, *FLJ20373*, *MS4A1*, and *ARHGEF39*; Naïve B cells Immunoglobulins Lambda Light chains (IGL)-high, characterized by the specific expression of *IGLV2-23*, *IGLV2-11*, *IGLV2-14, IGLC3*, *IGLC1*; Plasma cells, with *SDC1*, *IGHG3*, *IGJ*, *CD38*, and *XBP1* as cell-type markers and Plasmablasts, with the specific expression of *IGKV2-28*, *IGKV2D-28*, *CD5*, *IL2RA* and *FGR*) (Figure 3a, Figure S3). Interestingly, apart from the general B-cell lineage, we have managed to identify the specific B-cell population associated with response: the Naïve B-cell population, in particular the one characterized by overexpression of IGK (Naive B cells IGK-high) (Figure 3b, Table S7). In addition, we have identified three stromal immune cell types associated with bad response: Plasmacytoid Dendritic Cells, Macrophages and Gamma Delta T cells (Table S7).

### 2.8. Specific Stromal Cell Population Composition in the TME of Our Cohort of Metastatic Melanma Patents in Treament with Nivolumab

To obtain the cellular distribution in each of the patients treated with Nivolumab, two techniques were used to deconvolute the bulk RNA-seq derived expression into stromal cell types: one based on marker genes (MCP-counter) and the other based on the transfer of the bulk RNA-seq data to annotated single-cell data (CIBERSORTx).

MCP-counter-based computational cell type quantification reinforced the critical contribution of the B-cell lineage in the response to Nivolumab, among all cell types including T cells. Indeed, the B-cell lineage is the only cell type associated to response in our bulk RNA-seq data (Figure 3c). Moreover, the absolute B-cell score between good responders and bad responders is significantly different (Table S7). While B cells conform the dominant population among the good responders, in the severe bad responders (IMK37, IMK39), the B-cell score is close to null.

In order to increase the resolution of the B-cell lineage of our dataset, a complementary in silico cytometry method using cell subtypes annotated in single-cell RNA-seq data from ICB-treated melanoma patients as a signature matrix with a specialized version of CIBERSORT (CIBERSORTx) was applied over our bulk dataset (Figure 3d). Interestingly, the contribution of Plasma cells is associated with response to Nivolumab, and we observed an increasing trend of the Naïve B-cell population in good responders. None of the rest of the cell types were associated with response or resistance (Table S7).

### 2.9. Validation of the Bulk RNA-seq Transcriptomic Gene Signature Using Single-Cell RNA-seq Data

The cohort of ICB-treated melanoma patients whose tumors were sequenced at the single-cell level constitutes an extraordinary external Validation cohort for the bulk transcriptomics results. At the stromal cell population level, our hypothesis of the association of the B-cells lineage with response to anti-PD1 treatment was validated in this single-cell dataset, not only for B cells in general, but also for the refined B-cell-specific population associated with response that is the Naïve B-cell population, in particular the one characterized by overexpression of IGK (Naive B cells IGK-high) (Figure 3b, Table S7). Moreover, our bulk RNA-seq data show the association of the B lineage with response to Nivolumab, with a trend towards the subtype-specific association of Naïve B cells (Figure 3c,d, Table S7).

In an attempt to validate our signature at the gene level, we identified the common genes associated with response between the associated genes of the scRNA-seq dataset and our bulk RNA-seq results. Since this cohort was composed of patients that were treated with any of these ICB approaches: anti-PD1, anti-CTLA4 and anti-CTLA4 plus anti-PD1, we only selected the expression data for the patients treated with anti-PD1. In this setting, eight genes overlap with an adjusted *p*-value < 0.001 (*CD19*, *IGHM*, *CD22*, *IGHG3*, *IGHGP*, *IGHG2*, *POU2AF1* and *UPP1*). Most of the overlapping genes are associated with the B-cell lineage, constituting a gene-specific validation of our hypothesis (Figure 3e, Table S8).

Finally, we have investigated the conformation of Tertiary Lymphoid Structures using our bulk RNA-seq data in combination with the single-cell RNA-seq dataset. CXCR5 is the receptor for CXCL13, which is secreted at the tumor site to recruit B cells from circulation [32]. As this interaction is essential for the formation and organization of the TLS [32], CXCR5 is a marker of the presence of TLS in the tissue. Single-cell data shows that most clusters identified as B-cell populations were characterized by *CXCR5* expression, particularly Naïve B cells, and the IGK-high subtype and Plasmablasts (Figure 3f). Interestingly, the differential expression analysis in the single-cell RNA-seq dataset shows an association with response, while in our bulk RNA-seq that association does not reach statistical significance; it is only a trend (adj *p*-value < 0.1) (Figure 3f). Overall, this denotes the relevance of the expression of the TLS marker in the intra-tumoral B-cell populations for the response to ICB.

### 2.10. Validation of the B-Cell Signature by Multiplex Immunofluorescence

We set out to investigate the myeloid and lymphocytic contexture of 16 cutaneous metastatic melanomas of the Discovery Cohort in FFPE tissue samples using two complementary multiplex panels to enable the simultaneous examination of several cellular markers. The myeloid and lymphoid cell panel included the myeloid marker CD11b, the phagocytic cell marker CD68 for macrophages, CD3 and CD8 for T cells, CD20 for B lymphocytes and the melanocytic differentiation marker MELAN-A. The melanoma-associated B-cells panel included CD19, CD20 and CD138 for B-cells, Plasma cells and plasmoblasts.

**Figure 4:**
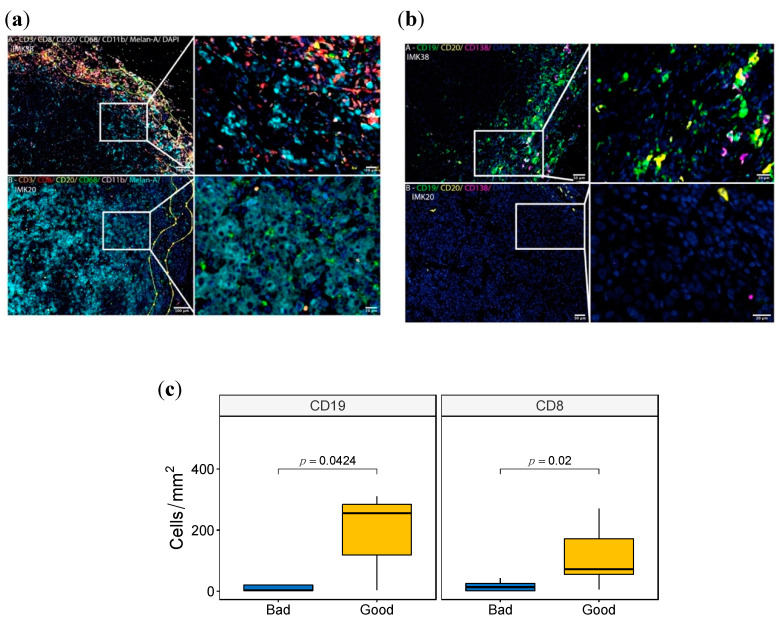
**Multispectral Immunofluorescence tissue imaging of good and bad responder cases.** (**a**,**b**) Fluorescence panels images of the markers CD11b, CD68, CD3, CD8, CD20, MELAN-A in (**a**) and CD19, CD20 and C138 in (**b**) of the patient IMK-20 (bad responder) and IMK-38 (good responder). (**c**) Boxplot with the Wilcoxon test comparing the density (Cells/mm^2^) between good and bad responders of CD8 and CD19.

Based on the fluorescence panels, cells were further subclassified as CD11b+, CD68+, CD3+, CD8+ and CD20+. For the myeloid and lymphoid cell panel, CD4+ T cells were defined as CD3+ CD8-. MELAN-A was used to visualize the melanoma cells. For the melanoma-associated B-cells panel, subpopulations were then classified as: (i) Total B cells (CD19+CD20-, CD19+CD20+ and CD19-CD20+ cells), (ii) Plasmablasts (CD19+CD20- CD138- cells), (iii) Plasma cell-like (CD19+CD20–CD138+ cells), and (iv) Mature Plasma Cells (CD19-/+CD20-CD138+ cells), as previously described [33]. Cells negative for these markers were defined as “other cell types”. Using these two complementary multiplex immunolabeling panels to simultaneously assess different markers in a single FFPE tissue section for each panel, we showed a variance between cases with good response (IMK38) and poor response (IMK20) (Figure 4, and Figure S4). Differences were observed in the distribution and density of CD3+ and CD8+ T cells, CD19+ and CD20+ B lymphocytes, the myeloid marker CD11b and the phagocytic cell marker CD68 within macrophages subpopulations. Significantly, more CD8+ T cells and CD19+ B lymphocytes were found in good responder cases (Figure 4c). In addition, more immune cell infiltration was seen in both the invasive margin and intra-tumoral region of good responders. Interestingly, intra-tumor lymphoid structures were observed in cases with good response. Moreover, CD8+ T and B-cell-rich areas were seen in intra-tumor lymphoid structures and associated with cases with good response (Figure 4a, Figure S4a). In an attempt to identify distinct intra-tumor B-cell subpopulations, we used multiplexed analysis to simultaneously detect the expression of CD19, CD20 and CD138 on B cells. We showed that Plasma cell-like cells (CD19+CD20–CD138+) and mature Plasma cells (CD19-/+CD20-CD138+) were more abundant in cases with a good response (Figure 4b, Figure S4b). This is in accordance with the association of Plasma cells with a good response to Nivolumab discovered in our bulk RNA-seq data.

In view of this, we tested the statistical correlation of the cell population enrichment as assessed by multispectral immunofluorescence and transcriptomics deconvolution. A high Spearman correlation between CD19 and CD20 markers by immunofluorescence and Naïve B cells from bioinformatic deconvolution validates the B lymphocyte abundance shown in the bulk transcriptomics data. Among all the comparisons, the pairing between Plasma cell-like density from the immunofluorescence analysis and Plasma cell score via deconvolution is the one with the highest correlation, statistically corroborating the intratumoral abundance of Plasma cell populations as a potential marker of response to Nivolumab (Table S9).

### 2.11. Higher Abundance and Diversity of BCR and HLA, but Not of TCR Clonotypes, Are Associated with Response to Nivolumab

V(D)J recombination consists of the assembly of the different genomic segments of the variable chains of the antigen receptors in both T and B lymphocytes (TCR, BCR). V(D)J recombination is responsible for generating the diversity needed for antigen recognition; in tern, HLA determines the presentation of the antigen from the dendritic cells in the priming phase of the adaptive immune response.

**Figure 5:**
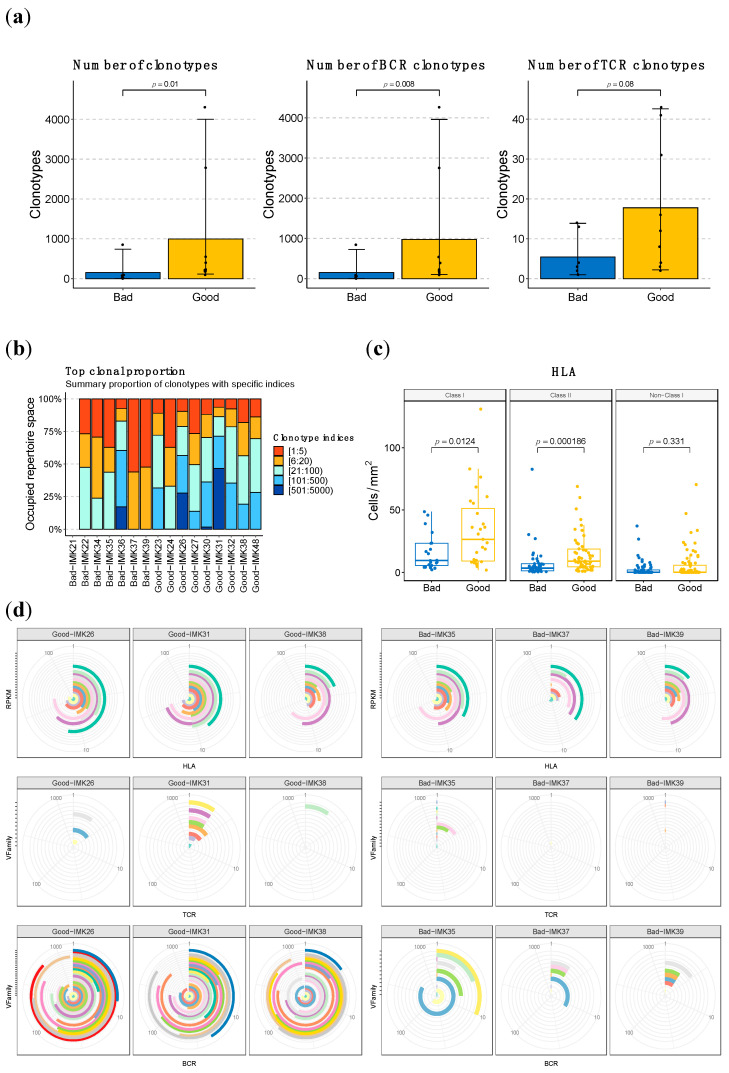
**HLA, TCR and BCR abundance and diversity is higher in good responders to Nivolumab.** (**a**) Quantification of the sum of BCR and TCR clonotypes in good vs. bad responders and based on the stratification by type of cell. (**b**) Clonal proportion and diversity estimation of clonotypes. (**c**) Quantification of the HLA loci in good vs. bad responders based on type of HLA. (**d**) Representative depictions of the abundance and diversity of HLA, and the VFamily of TCR and BCR clonotypes, based on bulk RNA-seq data. Each concentric circle and color represents a variant, and the covered angle of the circumference indicates the amount of the specific variant.

Given that the involvement of the B-cell lineage in the background and potential reactivation of the anti-tumor immune response has been outlined in multiple dimensions of our analysis, we decided to characterize the abundance and diversity of the BCR, TCR clonotypes and HLA loci (Figure S6), aiming to confirm the potential functional translation of an enhanced transcriptomic signature of the B cells. Consistently, we identified that the number of clonotypes in general (combined count of BCR and TCR) was significantly (*p*-value < 0.02) higher in good versus bad responders; in addition, the major contribution of B cells compared to T cells was again highlighted when we stratified by BCR and TCR (Figure 5a). In addition to the total amount, we found differences in the diversity of the clonotypes (Figure 5b). In good responders, the clonotype repertory is distributed with a similar proportion for the clonotypes of different frequencies. Nevertheless, in bad responders, most of the clonality is biased to the top clonotypes. Comparing the degree of clonal expansion by the D50 index, there is a significant difference in BCR response, where good responders present more diversity in BCR clonotypes.

**Figure 6:**
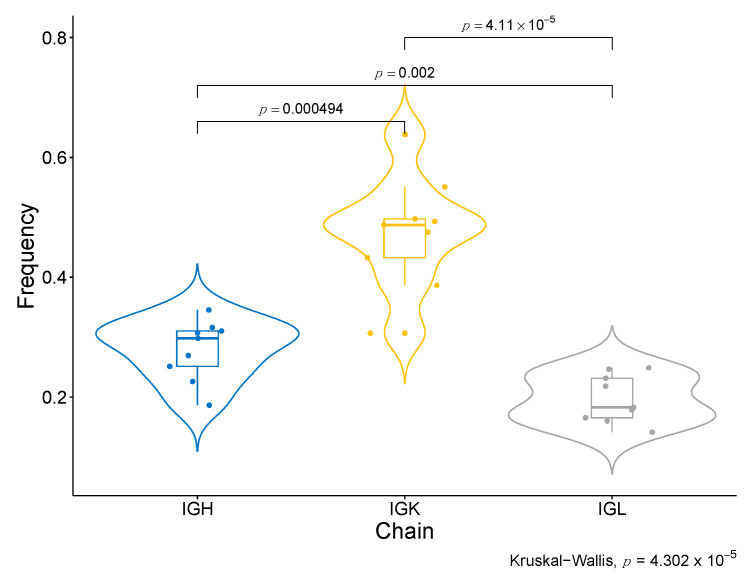
**BCR chain diversity among good responders.** Comparison of the abundance between BCR chains in good responders.

Moreover, we have identified specific BCR clonotypes that are more different between good and bad responders. The constant area of Kappa immunoglobulin light chains (IGKC), which is part of the transcriptomic signature of response to Nivolumab, is present in all of them (Table 1, Figure S5a). Moreover, all top clonotypes are composed of IGK (Table 1). When the abundance of each type of BCR chain in good responders is compared (Figure S7), the IGK type is considerably superior to the other types of chains, such as IGL and IGH (*p*-value <0.001) (Figure 6). These findings are coherent with the association of Naïve B cells IGK-high with response to ICB, as identified in the single-cell data analyses. Another interesting finding regarding the specific clonotypes associated with response is that the top 100 associated clonotypes are scarcely represented in the tumors of the severe bad responders that showed a zero score for the enrichment in B cells in the deconvolution analysis (IMK37, IMK39) (Table S10).

With regard to HLA loci abundance, we also evidence a role for it in the response mechanism in our patients, given the difference in the number of predicted loci between good responders and bad responders, which is particularly significant for the Class II loci (*p* < 0.001) (Figure 5c, Figure S5b).

When assessing the diversity in BCR, TCR, and HLA, we evidenced that, particularly for VFamily BCR, it is proportional to therapeutic response. In addition, we identified higher abundance and diversity in BCR compared to TCR, in line with the prominent implication of B lymphocytes that we have observed in the response to Nivolumab in our cohort. This pattern is exacerbated with regard to response, where bad responders have less abundant and diverse BCR and even lower levels of TCR than good responders (Figure 5d).

**Figure 7:**
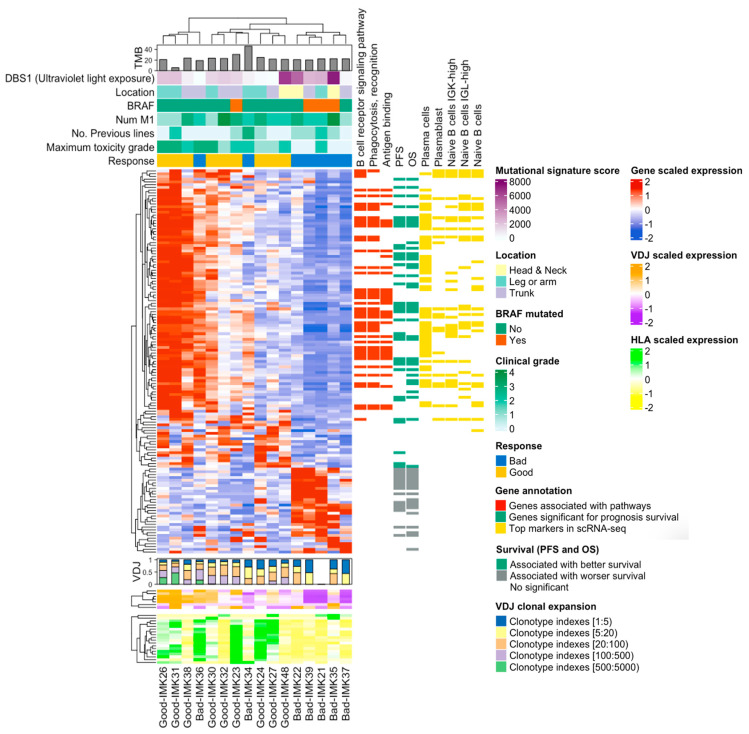
**Onco-heatmap: integration of bulk RNA-seq data, the VDJ and HLA abundance and genes features.** In the upper part of the ComplexHeatmap, we represent the clinical and genomic information. The DE expressed genes in good versus bad responders are located in the center of the plot. The diversity and abundance analysis of VDJ are plotted just below the DE analysis, using a bar plot and heatmap as graphs, respectively. HLA expression is presented at the bottom of the graph. Finally, details about the differentially expressed genes can be found on the right-hand side. We depict GO information, relationship with survival and gene validation in single-cell RNA-seq cohort.

Finally, the integration of the transcriptomic signature of response to Nivolumab with the VDJ and HLA abundance indicates that basal somatic recombination of VDJ and antigen presentation capacity are amongst the processes that are critical for the efficacy of the treatment (Figure 7).

### 2.12. Machine-Learning-Based Models Based on Our Transcriptomics Gene Signature Predict Response in Eight External Melanoma Cohorts Treated with ICB

Predictive gene signatures have been developed based on the variable importance of the Random Forest (RF) model. In our cohort, the predictive power of the gene signature was characterized by an AUC of 0.98, and the subset of selected genes were *DOK6*, *AC084082.1*, *IGKV1-27*, *IGLV3-1*, *CLEC4E*, *FRRS1L*, *IGHV6-1*, *IGKV3D-11*, *IGLV6-57*, *TNFRSF12A*, *AC051619.7*, *FDCSP*, *SYPL2*, *TNFRSF11B* and *ADAMTS9-AS1*. Interestingly, 11 of the 15 genes are also prognostic (Table S6), and 4 genes of this selected predictive signature are also associated with response to Nivolumab in the initial heterogeneous cohort composed of cutaneous, mucosal and ocular melanomas (Table S3).

In addition, given the small number of patients, we have carried out external validation with eight external cohorts of metastatic melanoma patients, six treated with anti-PD1, one treated with anti-PD1 + anti-CTLA4 and one treated with anti-CTLA4 in the context of five different studies [34,35,36,37,38] (Table S11).

**Figure 8:**
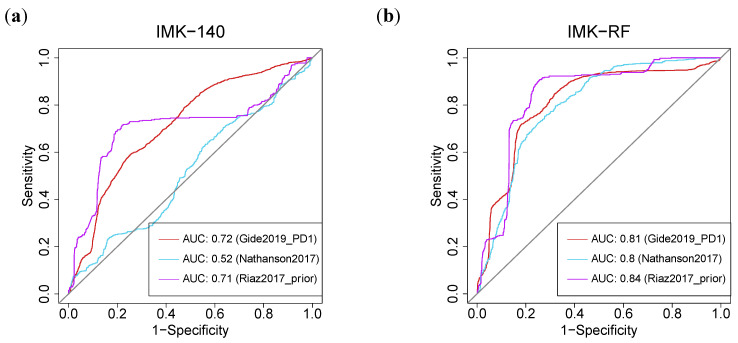
**Machine-Learning-based predictive power of the transcriptomic signature.** ROC curve for the (**a**) 140-genes model by cross-validation (CV) and (**b**) Random Forest (RF) model in three different external cohorts.

Interestingly, the AUC (Area Under the Curve) of our gene signature based on the 140 DE genes outperformed the published models [39]. Using our two models, we were able to improve the response prediction potential in seven of the eight cohorts (Table S11). In particular, using feature selection based on Random Forest (IMK-RF, 15 genes), our model outperformed the prediction of response with an AUC value of at least 0.8 in three of the cohorts [34,36,37]. In these three cohorts, both of our algorithms, the full model with 140 genes, or the model with 15 genes based on RF, yielded higher AUCs in comparison with the TIDE (Tumor Immune Dysfunction and Evolution) algorithm [40]: 0.81 (IMK-RF) and 0.72 (IMK-140) vs. 0.62 (TIDE) in (Gide et al., 2019—PD1) [34]; 0.82 (IMK-RF) and 0.51 (IMK-140) vs. 0.45 (TIDE) in (Nathanson et al., 2017) [36]; and 0.84 (IMK-RF) and 0.71 (IMK-140) vs. 0.23 (TIDE) in (Riaz et al., 2017—prior) [37] (Figure 8, Table S11).

## 3. Discussion

This study aims to address the absence of effective biomarkers of response to IT, specifically to IT for the inhibition of the PD1/PDL1 axis, in patients with metastatic melanoma. In order to accomplish this, we carried out a coding and non-coding transcriptomic analysis of FFPE samples from good and bad responders to Nivolumab. So far, few studies have used a transcriptomic approach to identify molecular prognosis or response predictors in patients subjected to this type of treatment, and the majority included a very discrete number of patients and cohorts that were heterogeneous in terms of subtype and ICB treatment and regimen [29]. To our knowledge, our approach that surveys All RNA species to identify Nivolumab response biomarkers has not been employed before. In addition, we are the first to generate and validate, in several cohorts, a response prediction model using machine-learning algorithms that selected a signature of 15 genes of high predictive value.

The evaluation of our whole Discovery cohort of 21 patients with metastatic melanoma, which included 16 cutaneous, 3 uveal and 2 mucosal melanomas, yielded a response-related transcriptome signature of 22 genes, with *ALDH1A2* as the most DE gene. *ALDH1A2* is the rate-limiting enzyme responsible for the catalysis of retinoic acid from retinaldehyde. It has been recently reported as a tumor suppressor gene in ovarian cancer, where it is amply downregulated, and this is related to bad prognosis [41]. When it comes to indication of response, *ALDH1A2* is upregulated in the bad responders of our cohort. This could be explained by the fact that the retinoic acid generated from *ALDH1A2* expressed in dendritic cells has been reported to work in conjunction with TGF-β to facilitate the development of Foxp3^+^ Treg cells in the intestine [42]. Therefore, *ALDH1A2* expression constitutes an interesting candidate for explaining and marking the inefficient action of Nivolumab, independent of the molecular differences that characterize these types of melanoma [43,44].

The restrictive analysis of the 16 cutaneous melanoma patients of the Discovery cohort led to the identification of 140 genes DE in the good responders to Nivolumab. Interestingly, the genes are mostly expressed in immune cells, particularly in the B-cell lineage. Pathway analysis mainly shows processes related to immune response, with high involvement of B cells. It is important to note that, in addition to the response predictive potential, we have also evidenced a correlation with the outcome of 55 and 59 of the 140 genes of the transcriptomic signature, including both tumor-related genes and B-cell-related genes associated with PFS and OS, respectively. Among them, the *IGKV4-1* gene, associated with both PFS and OS, codes for segment V of the variable domain of the immunoglobulin light chain, and is involved in antigen recognition; high expression of *IGKV4-1* is correlated with longer survival. Other B-cell-related genes with high expression associated with PFS and OS are *IGLV6-57* and *IGHV3-21*, coding for the V segment of the variable domain of immunoglobulin light chains, also participating in antigen recognition, and the *IGHA1* gene, encoding for the constant region of an immunoglobulin heavy chain and involved in antigen binding. Interestingly, those genes that are less expressed with better outcome are mainly tumor-related, including *LGR5,* which codes for a member of the Wnt signaling pathway and has been described as a cancer stem cell marker in colon cancer related to prognosis and to response to chemotherapy treatment in colon [45] and gastric cancer [46]. In addition, the increased expression of *LGR5* in the bad responders and its correlation with outcome could mark a higher de-differentiation, where the melanoma tumors express markers of neuroectodermal origin [47]. An additional prognostic variable of importance identified in our study is the presence of lung metastasis, which is associated with better PFS and OS. This is the first report of such a correlation. Interestingly, lung metastases, independent of the tumor origin, have a high immunogenic profile that could be related to the better outcome [48].

In order to relate the enhancement of the B-cell function in the good responders to Nivolumab to the function of BCR and HLA systems, in comparison with TCR, we evaluated the abundance and diversity of the BCR and TCR clonotypes and the HLA loci expressed by the patients in our cohort for their association with response. It is important to note that the gene coding for one of the constant regions of the immunoglobulin light chains, *Immunoglobulin Kappa Constant* (*IGKC*), a component of the prediction signature, together with specific variants of the IGK variable region, forms part of the top five most expressed clonotypes in good responders.

Strikingly, the contribution of both BCR and HLA abundance and diversity to the response to Nivolumab was exclusive and significant, which contrasts with the predominantly reported influence of T-cell signaling in the mechanism of resistance to Nivolumab [29,49,50]. In our cohort, TCR clonotypes were very scarce in terms of amount and diversity among the good responders. The decrease in TCR clonality has been previously referred to as acquired resistance to ICB in NSCLC due to the neoantigen loss associated with the treatment; however, it was also characterized by a specific T-cell expansion [26] that is not reflected in our analysis, probably due to the fact that our experimental design consisted of searching for single-point pretreatment biomarkers.

The identification of the relevant B-cell-signaling influence on the clinical benefit led us to characterize the composition of the stromal cells of the tumor microenvironment in search of specific subtypes that could shed more light on the mechanisms of the observed anti-tumor effect of the B-cell enrichment in the good responders of our cohort. Using deconvolution analyses, we could confirm such enrichment in the B-cell lineage, while identifying the most severe bad responders as the ones with the lowest B-cell enrichment scores. In addition, we could conclude that stromal Plasma cell type is the specific B-cell lineage population significantly enriched in the good responders of our cohort.

The biological validation of this B-cell-specific signature and the refined subsets was performed in a Validation cohort of 32 metastatic melanoma patients treated with ICB, for which single-cell RNA-seq data of the tumor samples had been generated. This approach led us to confirm the association of the B-cell lineage with response to anti-PD1. Moreover, we defined a specific B-cell subtype associated with good response to ICB treatment that, according to the top expression markers, we have named “Naïve B cells IGK-high”. Interestingly, there is a common denominator contributing to response in both the Discovery and Validation cohorts: high expression of the constant and specific variable regions of the Kappa light chains of IGs. IGKC has been defined as the first immune marker of response to cancer treatment in the context of chemotherapy in breast, non-small cell lung and colorectal cancer [51]. Here, we demonstrate its further implication in the response to ICB. A manifestation of the convergence of both B-cell subtypes is the expression of *IGKV1-39* as one of the top markers of the “Naïve B cells IGK-high” at the time of one of the IGK variants, together with *IGKC*, of the Top 5 BCR clonotypes of the good responders of the Discovery cohort. In addition, IGKC is known to be secreted by tumor-infiltrating CD138+ mature Plasma cells [52]. Accordingly, multispectral immunofluorescence on our metastatic melanoma patients shows that CD138+ Plasma-cell like and mature Plasma cells are more abundant in cases with good response, and correlate with the transcriptomics data. Interestingly, not only the transcript expression, but also the protein expression of stromal Plasma cell-produced IGKC has been associated with prognosis in several solid tumor types [52,53,54].The importance of the B cells in modulating anti-tumor immune responses has just recently begun to be understood. B cells and Plasma cells have effector activities that can activate the complement cascade and engage in antibody-dependent cell cytotoxicity (ADCC). Moreover, B cells are part of the Tertiary lymphoid Structures (TLS), which are well-organized, non-encapsulated structures of immunological and stromal cells. The development of this lymphoid neogenesis process in peripheral tissues is associated with a better response to immunotherapy. The TLS offers a region of strong B-cell antigen presentation that can promote the best T-cell activation, effector functions and the production of effector B cells, which can then be further differentiated into memory B cells or antibody-secreting Plasma cells [55]. The interaction between B cells and antigen-exposed and worn-out CD8+ T cells within mature TLS is of clinical significance, since it has recently been linked to a better immune checkpoint blockade (ICB) response in melanoma [56].

In addition to the knowledge important for response mechanistic deciphering and eventual intervention, the short-term clinical use of the transcriptomic signature was assessed via the construction of machine learning-based prediction models derived from the transcriptomic signature identified in this study. Our proposed model outperformed the only reported prediction model algorithm, the TIDE score. In addition, the RF-based model permitted the reduction of the 140 genes signature into a signature composed of 15 genes, where the role of immunoglobulins remains of importance. Interestingly, 11 of these genes are also prognostic and 4 are associated with response in all types of melanoma. These models were also validated in eight reference cohorts confirming the capacity of our gene signature to predict the response to ICB in melanoma patients.

In summary, integrative analysis of the data of our study indicates that the basal expression of a gene signature enriched in B-cell-related genes and the presence of intra-tumoral Plasma cells with high expression of IGKC can predict the promotion of the anti-tumor immune response in the PD1 blockade scenario in melanoma patients with greater potential than the reference prediction algorithm. This is a groundbreaking finding in the field that tallies with our hypothesis of the best predictive biomarkers being the ones at the interaction of the tumor and the extended immune system beyond T lymphocyte markers.

## 4. Materials and Methods

### 4.1. Subjects

A Discovery cohort of 21 metastatic melanoma (16 cutaneous, 3 uveal, 2 mucosal) patients treated with Nivolumab donated FFPE tumor biopsy samples that were collected prior to treatment (Table S12). All analyses posterior to the transcriptomic profiling of response were focused on cutaneous melanoma patients, given the specificity of the transcriptomic signature corresponding to the homogeneous group of cutaneous melanoma patients.

Patient biopsies were selected to exclude lymph node metastases when possible, and to include only samples with availability of clinical data and information on progression after treatment. The cohort is distributed into good responders (11) and bad responders (10), where the distinction criteria has been adapted from our previous study [22] to select extreme good and bad responders:

- Good responders: patients with maintained partial or complete response for a year or in treatment during at least one year.
- Bad responders: progression in less than 3 months from the start of IT. Of these, a subgroup of “severe” bad responders was defined as those who progressed in fewer than 60 days.

Response to Nivolumab was assessed according to “Response Evaluation Criteria in Solid Tumors” (RECIST v1.1 guide). The study follows the Declaration of Helsinki and has been submitted and approved by Comité de Ética de la Investigación Provincial de Málaga. The approval date was 26 October 2017, and the title of the research project was “Omics integration for precision cancer immunotherapy” (799818, H2020-MSCA-IF-2017). All patients signed an Informed Consent to participate in the study and received an information sheet about the project.

For the validation cohort, we employed a cohort of 32 melanoma patients treated with anti-PD1 and analyzed with single cell RNA-seq reported in Sade-Feldman, M. et al., 2018 [30].

### 4.2. Nucleic Acid Extraction

The tumor-specific area in FFPE melanoma samples was predefined by a pathologist. Two to four 10 µm slides were dissected for nucleic acid extraction, using the microtome HM 340E (Thermo Scientific, Waltham, MA, US). RNA was extracted with the RNeasy FFPE kit following the manufacturer’s instructions (Qiagen, Düsseldorf, germany; Ref. 73504).

### 4.3. Technical Validation

The technical validation of the All RNA-seq data was performed using real-time quantitative PCR (RT-qPCR) of the following selected genes based on the gene expression patterns and the representation of tumor and immune system candidate biomarkers: *TNFRSF11B*, *IGLV6-57*, *IGHA1* and *GRIA1*. RNA from selected samples of the Discovery cohort presenting high and low expression for the genes of the study was retrotranscribed into cDNA and subjected to RT-qPCR. The housekeeping control gene ACTB was used for normalization.

### 4.4. Next Generation Sequencing

RNA-Seq libraries were prepared using TruSeq Stranded Total RNA Gold (Illumina; Ref. 20020598) and indexed by IDT for Illumina—TruSeq RNA UD Indexes (Illumina; Ref. 20020591). These libraries include coding and non-coding RNA via ribosomal RNA depletion. In order to obtain better exclusion of ribosomal RNA, the manufacturer’s protocol was modified to include a double depletion. Libraries concentration (0.1–1 micrograms) was determined using a Qubit dsDNA BR kit, and the size distribution was examined by Agilent Tapestation 2200. The libraries each contained 0.1-Paired-end reads (75 bp × 2) acquired from the Illumina NextSeq 550 platform according to the corresponding protocol.

### 4.5. Bioinformatic Analysis

Fastq quality control was performed with FastQC. Fastq files were trimmed using the tool HISAT2 (v 2.1.0) with a customized index built using combined rRNA data from HGNC, ENA, SILVA (from the Latin silva, forest), and additional manually curated sequences from NCBI. Trimmed fastq files were mapped against the reference (genome build GRCh38) using STAR (v 2.5.1b) and read quantification was performed with the same tool. Percentages of uniquely mapped reads and M mapped reads were computed with Qualimap.

To perform the normalization and test for differentially expressed (DE) genes, we used the Bioconductor package DESeq2. A gene was considered DE if the baseMean count was >10, the absolute log2FC was >1.5, and the adjusted *p*-value was <0.05.

Pathway analysis was performed with R in-house scripts using two different approaches: gene set enrichment analysis (GSEA and DAVID) and network-based pathway analysis. The packages used were STRINGdb, clusterprofiler, pathfindR. We utilized MIXCR and Seq2HLA for HLA, TCR and BCR profiling. Somatic mutations were detected by GATK Mutec2 variant calling after STAR alignment. Additionally, a pipeline with picards, bedtools and vcftools was used. Tumor mutational burden (TMB) was defined as the total number of somatic mutations per coding area of covered tumor genome. We also evaluated 11 DBS COSMIC (v3.2) mutational signatures in our cohort. For validation in a scRNA-seq cohort, the dataset of anti-PD1 treated melanoma patients that conformed our Validation cohort was integrated at the Seurat environment, normalized and preprocessed. Next, we used PCA (Principal Component Analysis) in combination with UMAP (Uniform Manifold Approximation and Projection), together with the PanglaoDB markers to identify and distribute the cell populations defined by the scRNA-seq built expression dataset. Shared nearest neighbor modularity optimization combined with Louvain clustering algorithm was employed to identify clusters of cells. To increase the resolution of B-cell lineage, the number of communities was refitted. Finally, we localized the 140 DE genes of our bulk-seq data within the generated cell-type map and tested their overlap with the response-associated genes of the scRNA-seq dataset. Top marker genes were determined as differentially expressed genes ranked by adj *p*-values derived from cell-type comparisons. MPC Counter was used to infer the abundance of different immune cells populations of the tumor infiltrate using the normalized counts of RNA-seq, whereas CIBERSORTx, using the signature matrix produced by the single cell analysis, enabled us to increase the B lineage resolution.

### 4.6. Statistical Analysis

A likelihood ratio test was used to identify associations between clinical variables and the response variable 3 months after treatment.

The Wilcoxon test was used to compare clonotypes and HLA counts in good and bad responders. When comparing the frequency of the different BCR chains, the Kruskal–Wallis test was used. The diversity of BCR clonotypes in each sample was measured based on the D50-index. The D50-index was calculated by determining the cumulative frequency of total sequences that constitute 50% of the cumulative unique sequence frequency, and then compared among responses to identify differences.

Two different tests were used to identify the correlations between two variables: Pearson’s test to assess the linear relationship between TPM values from RNA-seq and ΔCt values from TaqMan, and Spearman correlation to identify monotonic relationships when comparing immunofluorescence density from multiplex immunofluorescence and the deconvolution proportion inferred from RNA-seq.

Survival analysis was performed with the Kaplan–Meier test. The log-rank test implemented in the R package survival (http://coin.r-forge.r-project.org; accessed on 1 June 2022) was applied to assess statistical significance in prognosis. Quantile stratification of the gene expression was used to group in high, medium and low categories.

Leave-one-out repeated cross-validation was the learning algorithm used for internal validation and for external validation. Random Forest was applied over the 140 DE genes to identify the gene signature. Gene selection is based on the importance of variables in the random forest during internal validation. For external cohort validation, the TIDE implemented in [40] was used as a comparison reference for the predictive power of our gene signature.

### 4.7. Multiplex Immunofluorescence

Multiplex immunofluorescence (IF) development and validation workflow and protocols were implemented as previously described [57,58]. Briefly, 4-micron sections of formalin-fixed paraffin-embedded (FFPE) tissue from the 16 patients of the Discovery cohort that presented sufficient material were deparaffinized, and antigen retrieval was performed using DAKO PT-Link heat-induced antigen retrieval with low pH (pH 6) or high pH (pH 9) target retrieval solution (DAKO). Depending on the multiplex immunofluorescence protocols, each tissue section was subjected to three or six successive rounds of antibody staining, each round consisting of protein blocking with 20% normal goat serum (Dako, Santa Clara, CA 95051, USA) in phosphate-buffered saline (PBS), incubation with primary antibody, biotinylated anti-mouse/rabbit secondary antibodies and Streptavidin-HRP (Dako, Santa Clara, CA 95051,USA), followed by TSA visualization with opal fluorophores (Akoya Biosciences, Marlborough, MA, USA) diluted in 1X Plus Amplification Diluent (Akoya Biosciences, Marlborough, MA, USA).

The myeloid and lymphoid cell panel included: CD11b (Rabbit monoclonal, clone EPR1344, 1:1000, Abcam (Cambridge, UK), product number ab133357), CD68 (Mouse monoclonal, clone PG-M1, ready-to-use, Agilent, Santa Clara, CA 95051, USA, product number IR613), CD3 (Rabbit polyclonal, IgG, ready-to-use, Agilent, Santa Clara, CA 95051, USA, product number IR503), CD8 (Mouse monoclonal, clone C8/144B, ready-to-use, Agilent, Santa Clara, CA 95051, USA, product number GA62361-2), CD20 (Mouse monoclonal, IgG2α, clone L26, ready-to-use, Agilent, Santa Clara, CA 95051, USA, product number GA604) and MELAN-A (Mouse monoclonal, clone A103, ready-to-use, Agilent, Santa Clara, CA 95051, USA, product number IR63361).

The melanoma-associated B-cells panel included: CD19 (Mouse monoclonal, clone LE-CD19, ready-to-use, Agilent, Santa Clara, CA 95051, USA, product number GA656), CD20 (Mouse monoclonal, IgG2α, clone L26, ready-to-use, Agilent, Santa Clara, CA 95051, USA, product number GA604), CD138 (Mouse monoclonal, IgG1, clone MI15, 1:100, Agilent, Santa Clara, CA 95051, USA, product number M7228). In the last round, nuclei were counterstained with spectral DAPI (Akoya Biosciences, Marlborough, MA, USA) and sections mounted with Faramount Aqueous Mounting Medium (Dako, Santa Clara, CA 95051, USA).

### 4.8. Tissue Imaging, Spectral Unmixing and Phenotyping

Each whole-tissue section was scanned on a Vectra-Polaris Automated Quantitative Pathology Imaging System (Akoya Biosciences, Marlborough, MA, USA). Tissue imaging and spectral unmixing were performed using inForm software (version 2.4.8, Akoya Biosciences, Marlborough, MA, USA), as previously described (PMID: 32591586; + Diego’s paper—no PMID yet). Image analysis was then performed in the whole-tumor area (referred to as the total melanoma area) using the open source digital pathology software QuPath version 0.2.3, as previously described [58]. In short, cell segmentation based on nuclear detection was performed using the StarDist 2D algorithm, a method that localizes nuclei via star-convex polygons, incorporated into QuPath software by scripting. Invasive margin was defined as a region of 100 µm width assessed in the interface tumor and stromal compartments (determined by cell marker MELAN-A).

A Random Tree algorithm classifier was trained separately for each cell marker by an experienced pathologist (CEA), who annotated the tumor regions. Interactive feedback on cell classification performance was provided during training in the form of image markup, significantly improving the accuracy of machine-learning-based phenotyping. (PMID: 29203879, PMID: 32591586). All phenotyping and subsequent quantifications performed were blinded to the sample identity. Cells close to the border of the images were removed to reduce the risk of artefacts.

### 4.9. Special Case

We wish to note additional clinical information about patient IMK36. While this patient fulfills our criteria for bad responders, all the analyses indicate that he/she is an outlier. Soon after the start of the Nivolumab treatment, the patient presented ulcers in the legs and received antibiotic and corticoid treatment that could have inhibited the initial anti-tumor immune response. However, he/she has not been removed from the study in order to not reduce the sample size.

## Figures and Tables

**Table 1:** Top 5 BCR clonotypes enriched in good responders to Nivolumab.

Good Responders Count	Bad Responders Count	Clonotype Composition
1555	87	IGKV3-20, IGKJ1, IGKC
1364	8	IGKV1-33, IGKJ4, IGKC
917	129	IGKV1-39, IGKJ2, IGKC
818	49	IGKV1-5, IGKJ1, IGKC
816	47	IGKV3-15, IGKJ2, IGKC

## Data Availability

The data that support the findings of this study are available from the corresponding author, IB, upon reasonable request.

## Supplementary Materials

The following supporting information can be downloaded at: https://www.mdpi.com/article/10.3390/ijms23169124/s1.
