# Supplementary material for: High IGKC-Expressing Intratumoral Plasma Cells Predict Response to Immune Checkpoint Blockade
Source: Int J Mol Sci. 2022 Aug 15;23(16):9124. doi: 10.3390/ijms23169124 (PMC9408876; doi:10.3390/ijms23169124)
Supplement: Supplementary file 1 [file ijms-23-09124-s001.zip › ijms-1821643-supplementary.pdf]

SUPPLEMENTARY FIGURES AND TABLES

Figure S1

(**a**)

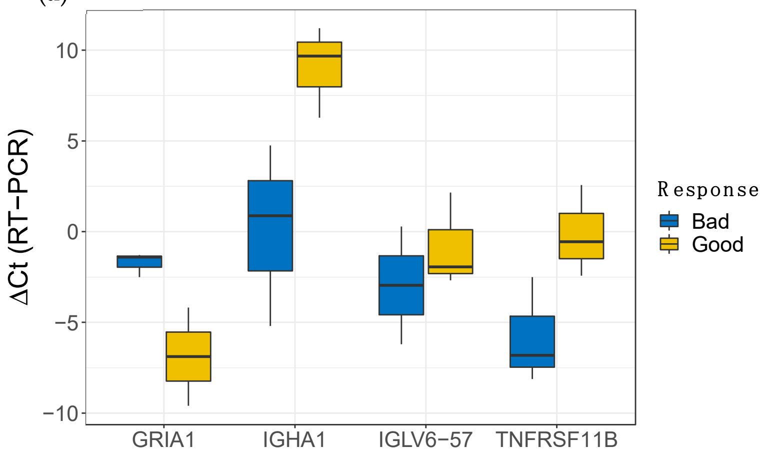

(**b**)

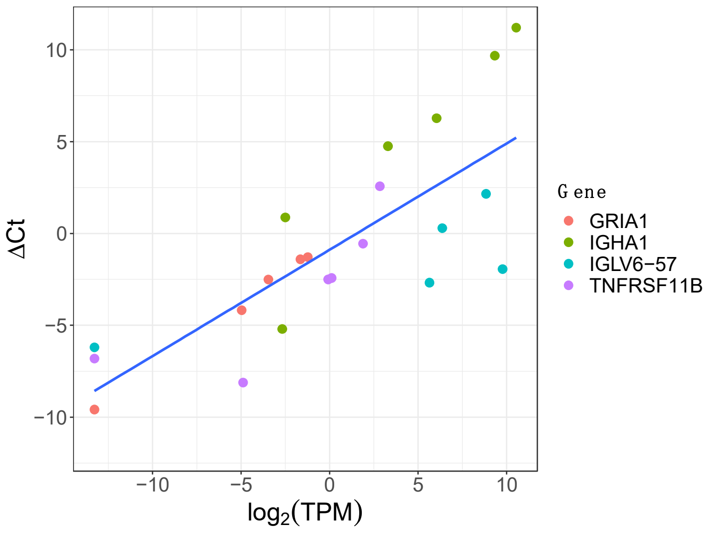

Figure S1. Correlation between RNA-seq and Taqman. (a) Boxplot with △Ct values from TaqMan (y-axis) for each gene (x-axis) grouping by response. **(b)** The scatter plot shows the log2 values of the Transcripts Per Kilobase Million (TPM) on the x-axis and the △Ct values from TaqMan (y-axis). A linear correlation is fitted with the confidence intervals.

Figure S2

(**a**)

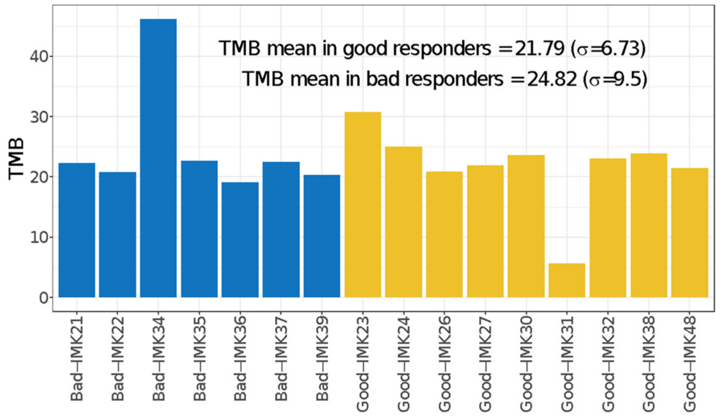

(**b**)

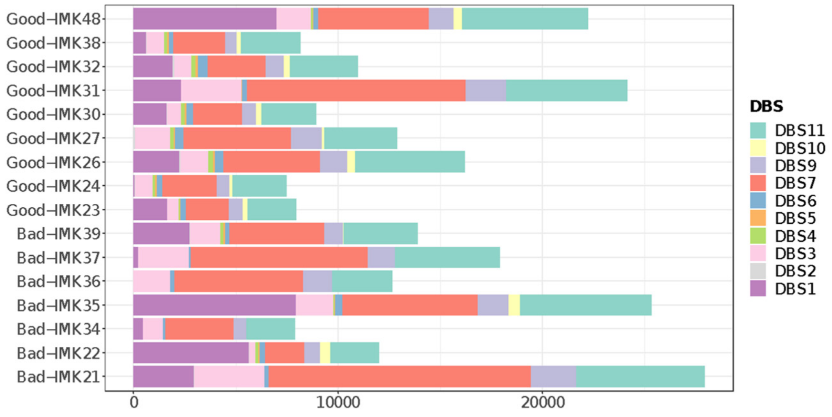

Figure S2. TMB and somatic mutational signature quantification and comparison in the cutaneous metastatic melanoma patients. (a) Bar plot per sample with TMB as height. Blue and yellow stratified the response by bad responders and good responders. Mean and standard deviation (σ) was computed for each group. **(b)** Doublet base substitutions are mutational signatures annotated in COSMIC. DBS1 {Ultraviolet light exposure}, DBS2 {Tobacco smoking, other mutagens, and clock-like}, DBS3 {polymerase epsilon exonuclease domain mutations}, DBS4 {unknown, correlated with clock-like} DBS5 {platinum chemotherapy treatment}, DBS6 {unknown}, DBS7 {defective DNA mismatch repair}, DBS8 {unknown}, DBS9 {unknown}, DBS10 {defective DNA mismatch repair} and DBS11 {possibly related to APOBEC mutagenesis}.

Figure S3

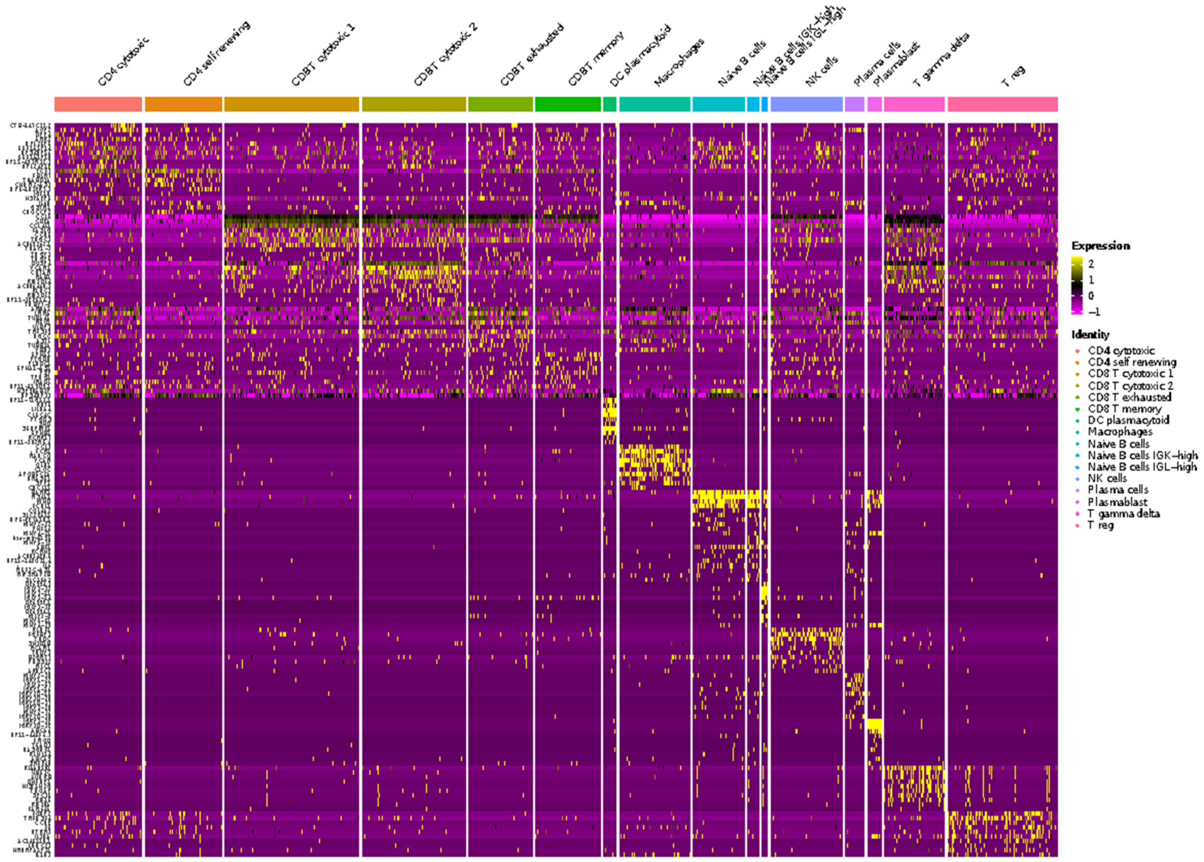

Figure S3. Expression marker heatmap based on scRNA-seq. Top 10 genes markers based on adj p-value for each cell line type established from the single cell RNA-seq analysis. Top markers were identified by differential expression analysis between cell types.

Figure S4

(**a**)

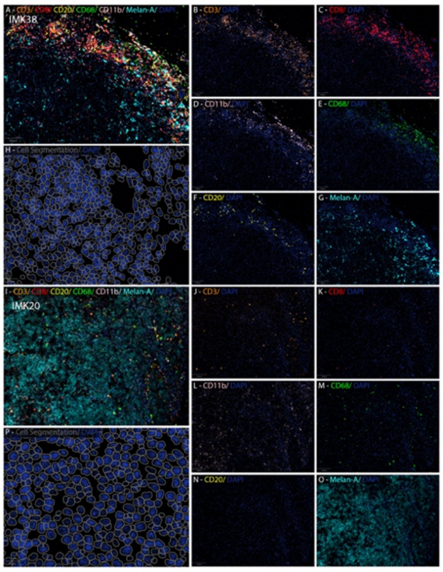

(**b**)

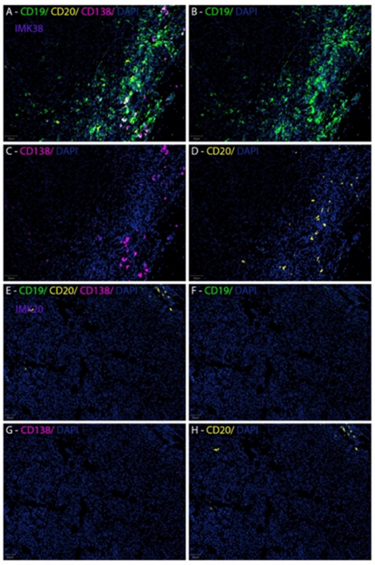

Figure S4. Multispectral Immunofluorescence tissue imaging comparing good and bad responder cases stratified by fluorescence panels. Fluorescence panels images of each of the markers CD11b, CD68, CD3, CD8, CD20, MELAN-A in **(a)** and CD19, CD20 and C138 in **(b)** of the patient IMK-20 (Bad responder) and IMK-38 (Good responder).

Figure S5

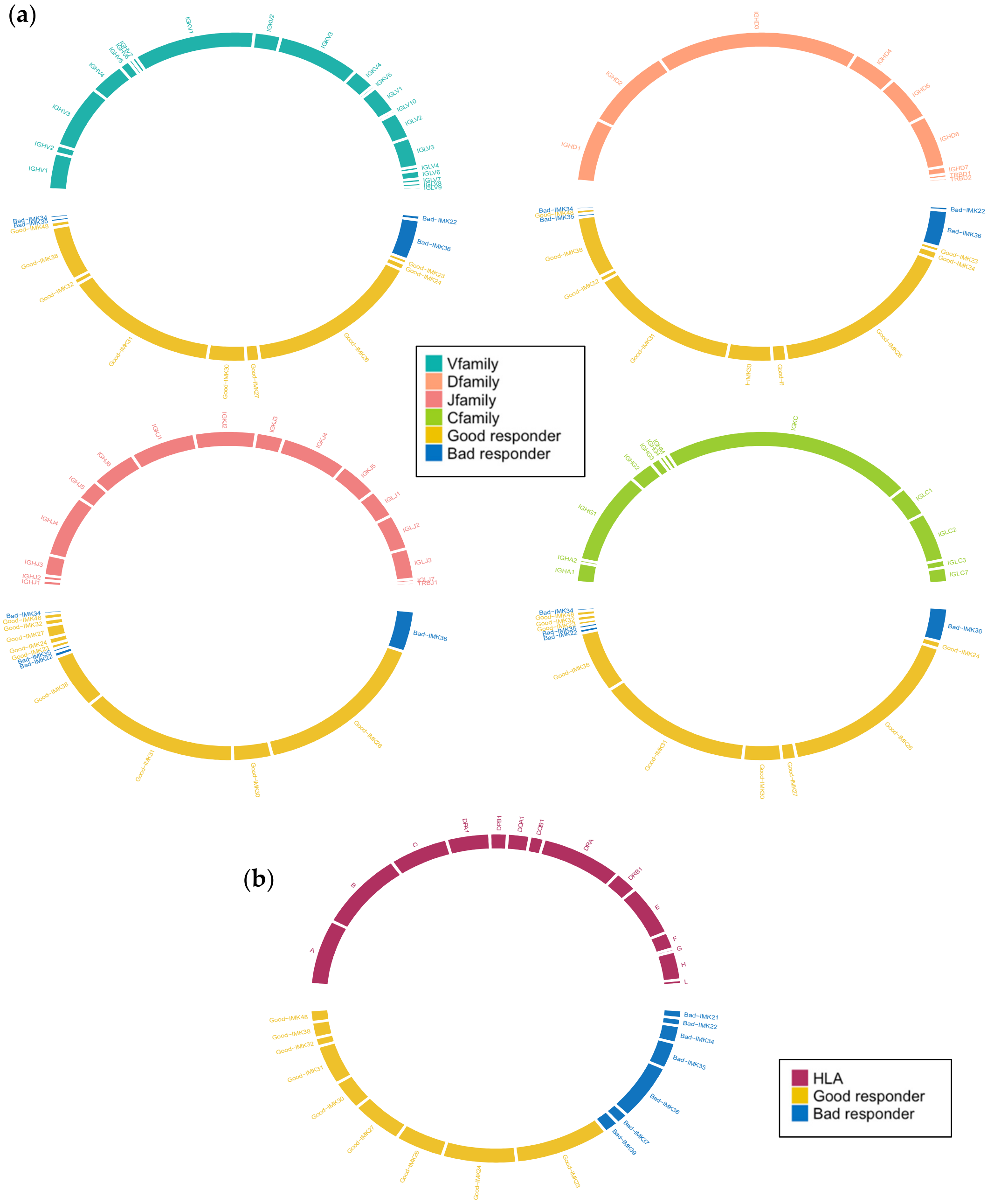

Figure S5. Diversity of the BCR families and HLA loci per patient. (a) The connecting lines indicate the BCR clonotypes with each patient presents. The width of the line indicates the total amount of the clonotype. Each plot depicts a different BCR family: Vfamily, Dfamily, Jfamily and Cfamily. The patients in yellow are the good responders, and in blue, the bad responders. **(b)** The connecting lines indicate the amount of HLA loci that each patient presents. Depicted in yellow, the good responders, and in blue, the bad responders.

Figure S6

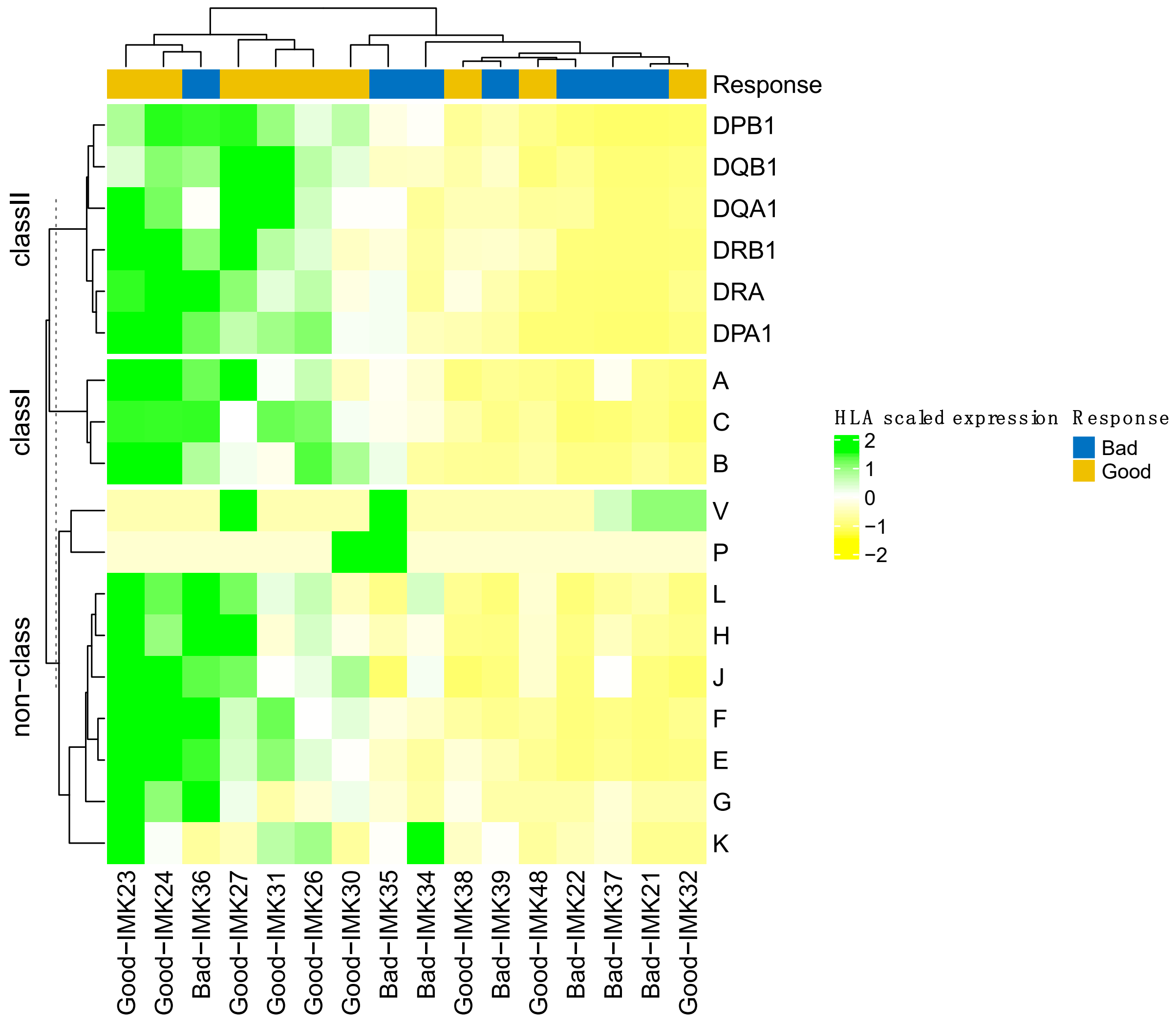

Figure S6. HLA heatmap. Heatmap with the expression values of HLA Class I, Class II and non-Class loci. Patients were ranked by a hierarchical unsupervised algorithm based on scaled expression.

Figure S7

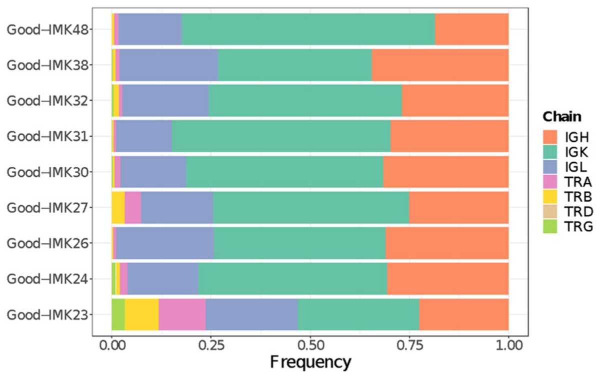

Figure S7. Diversity of the BCR/TCR chains in good responders. Distribution of BCR and TCR chain in good responders.

Table S1. Univariate analysis of the clinicopathological variables.

Variables	Likelihood ratio Test		Logrank p-value OS Survival		Logrank p-value PFS Survival	
	Melanoma cohort	Cutaneous melanoma cohort	Melanoma cohort	Cutaneous melanoma cohort	Melanoma cohort	Cutaneous melanoma cohort
**Age at start IT**	0.7022	0.3063	0.8268	0.1071	0.8544	0.2548
**Sex**	0.8188	1	0.697	0.8784	0.7858	0.9696
Stage at diagnosis	*0.0109*	*0.0573*	*0.0358*	*0.0708*	*0.0389*	*0.1376*
**Diabetes**	1	1	0.5512	0.5692	0.525	0.5805
**Dyslipidemia**	0.2105	0.2125	0.4015	0.4269	0.2844	0.2792
**Hypertension**	0.3698	0.1262	0.401	0.0864	0.3735	0.1215
**BRAF**	0.3034	0.2615	0.1781	0.45236	0.2204	0.4589
Lung metastasis	*0.0075*	*0.035*	*0.0015*	*0.0036*	*0.0038*	*0.0166*
Lymph node metastasis	*0.387*	*0.1451*	*0.0804*	*0.0395*	*0.231*	*0.1255*
**CNS metastasis**	1	1	0.3663	0.2434	0.4043	0.2918
**No. metastasis**	0.8272	0.683	0.8274	0.6751	0.5292	0.4021
**LDH first metastasis**	0.1645	0.1645	0.9178	0.9178	0.4637	0.4637
**LDH previous to IT**	0.6842	0.5368	0.9436	0.7277	0.6274	0.7277
**Lymphocytes previous to IT**	0.2271	0.2049	0.0583	0.0533	0.1825	0.9654
**Neutrophiles previous to IT**	0.4265	0.385	0.9606	0.273	0.8281	0.5646
**Platelets previous to IT**	0.1393	0.2474	0.7643	0.7641	0.1845	0.1108
**No. Previous lines**	0.2423	0.2559	0.1344	0.2073	0.3041	0.4024
IT toxicity	*0.0237*	*0.1058*	*0.0133*	*0.0858*	*0.0225*	*0.156*
**Maximum toxicity grade**	0.4545	0.7	0.1848	0.2339	0.6754	0.5217

The cohort was analyzed utilizing the cutaneous, ocular, and mucosal histological subtypes (melanoma cohort), and on the other hand using only the cutaneous subtype (cutaneous melanoma cohort). The Likelihood ratio Test was used to do a univariate analysis based on the dichotomous 3-month response variable: good and bad responder. Overall survival (OS) and progression-free survival (PFS) have been used in the logrank test.

Table S2. Univariate Cox proportional hazards regression analysis of OS and PFS.

Variable	Category	Melanoma (OS)		Cutaneous melanoma (OS)		Melanoma (PFS)		Cutaneous melanoma (PFS)	
		HR (CI %)	p-value	HR (CI %)	p-value	HR (CI %)	p-value	HR (CI %)	p-value
**Age at start IT**		1.0006 (0.9663 - 1.0361)	0.9725	0.9923 (0.961 - 1.0246)	0.6363	0.9996 (0.9676 - 1.0327)	0.9814	0.9913 (0.9628 - 1.0208)	0.56
**Sex**	*Female (Ref)*								
	Male	1.2321 (0.43 - 3.5298)	0.6976	1.0979 (0.3315 - 3.636)	0.8785	1.151 (0.4152 - 3.1907)	0.7869	0.978 (0.3118 - 3.0678)	0.9696
**Stage at diagnosis**	*I (Ref)*								
	II	2.0166 (0.2182 - 18.6337)	0.5364	2.0428 (0.2212 - 18.861)	0.5288	1.4249 (0.1583 - 12.8284)	0.7521	1.4471 (0.1607 - 13.0288)	0.7417
	III	1.0166 (0.1049 - 9.8558)	0.9887	1.0522 (0.1084 - 10.217)	0.965	1.0225 (0.1057 - 9.8931)	0.9847	1.0544 (0.1089 - 10.2122)	0.9635
	IV	7.8907 (0.7907 - 78.7385)	0.0784	9.291 (0.7787 - 110.8539)	0.078	7.6559 (0.7518 - 77.9631)	0.0856	6.6925 (0.5821 - 76.9498)	0.1271
**Diabetes**	*No (Ref)*								
	Yes	0.5431 (0.0707 - 4.1722)	0.5573	0.5539 (0.0703 - 4.3616)	0.5747	0.5326 (0.0698 - 4.0609)	0.5433	0.565 (0.0726 - 4.3978)	0.5856
**Dyslipidemia**	*No (Ref)*								
	Yes	0.5278 (0.1158 - 2.4055)	0.4089	0.5364 (0.1128 - 2.5504)	0.4337	0.4463 (0.0989 - 2.013)	0.2938	0.4365 (0.0935 - 2.0386)	0.2918
**Hypertension**	*No (Ref)*								
	Yes	0.5952 (0.2065 - 1.7153)	0.3366	0.308 (0.0906 - 1.0468)	0.0592	0.7055 (0.2542 - 1.9579)	0.503	0.4068 (0.128 - 1.2926)	0.1273
**BRAF**	*No (Ref)*								
	Yes	2.1927 (0.6796 - 7.0747)	0.189	1.6607 (0.4362 - 6.3226)	0.4571	2.0341 (0.6416 - 6.4488)	0.2278	1.6357 (0.4391 - 6.0932)	0.4633
**Lung metastasis**	*No (Ref)*								
	Yes	0.18 (0.0559 - 0.5801)	0.0041	0.1726 (0.0464 - 0.6418)	0.0087	0.224 (0.0762 - 0.658)	0.0065	0.2586 (0.0797 - 0.839)	0.0243
**Lymph node metastasis**	*No (Ref)*								

	Yes	2.7182 (0.8463 - 8.7299)	0.093	4.5411 (0.9508 - 21.6883)	0.0579	1.8915 (0.6443 - 5.5531)	0.2461	2.6982 (0.7213 - 10.0934)	0.1403
**CNS metastasis**	*No (Ref)*								
	Yes	2.0185 (0.4262 - 9.5597)	0.3761	2.5199 (0.5046 - 12.5849)	0.26	1.9221 (0.4128 - 8.9501)	0.4051	2.2844 (0.4708 - 11.0855)	0.3053
**No. Metastasis**		0.9316 (0.5484 - 1.5827)	0.7933	0.9136 (0.513 - 1.6271)	0.7589	1.0061 (0.6158 - 1.644)	0.9805	0.9995 (0.5902 - 1.6926)	0.9986
**LDH first metastasis**		0.9974 (0.989 - 1.0059)	0.5437	0.9974 (0.989 - 1.0059)	0.5437	0.9985 (0.9921 - 1.0048)	0.6354	0.9985 (0.9921 - 1.0048)	0.6354
**LDH previous to IT**		0.9996 (0.9952 - 1.0041)	0.8696	0.9998 (0.9952 - 1.0044)	0.9258	0.9999 (0.9958 - 1.0039)	0.9461	1 (0.9958 - 1.0043)	0.9969
**Lymphocytes previous to IT**		0.9989 (0.9978 - 1)	0.0564	0.9988 (0.9976 - 1.0001)	0.0614	0.9991 (0.998 - 1.0001)	0.0849	0.999 (0.9978 - 1.0001)	0.0857
**Neutrophiles previous to IT**		1.0001 (1 - 1.0003)	0.1365	1.0001 (1 - 1.0003)	0.0837	1.0001 (0.9999 - 1.0002)	0.3792	1.0001 (0.9999 - 1.0002)	0.3199
**Platelets previous to IT**		1.0001 (1.0000 - 1.0001)	0.0708	1.0001 (0.9999 - 1.0001)	0.4784	1.0001 (1.0001 - 1.0002)	0.0489	1.0001 (0.9999 - 1.0001)	0.2607
**No. Previous lines**		1.4154 (0.798 - 2.5105)	0.2348	1.4243 (0.7728 - 2.6249)	0.2569	1.2717 (0.7121 - 2.2712)	0.4165	1.2933 (0.6918 - 2.4176)	0.4204
**Toxicity IT**	*No (Ref)*								
	Yes	0.2755 (0.0931 - 0.8157)	0.0199	0.3443 (0.0969 - 1.2235)	0.0993	0.3067 (0.1059 - 0.8883)	0.0294	0.4233 (0.1248 - 1.4364)	0.1679
**Maximum toxicity grade**		1.8851 (0.7112 - 4.9967)	0.2024	2.0736 (0.7163 - 6.0028)	0.1787	0.9489 (0.3966 - 2.2701)	0.9061	0.9428 (0.3743 - 2.375)	0.9006

Table S3. 22 genes DE signature in good responders of all types of metastatic melanoma and 140 DE genes in good responders of metastatic cutaneous melanoma.

DE all types of melanoma		
**Gene**	**log2FoldChange**	**Adj p value**
ALDH1A2	-4.240	0.0008
EMCN	-2.478	0.010
FRRS1L	-4.068	0.010
PDE8B	-2.589	0.014
OPRD1	4.854	0.014
MMP3	5.453	0.014
ADAMTS9-AS1	-3.419	0.014
SYPL2	-2.020	0.016
LRFN5	-2.967	0.019
HPSE2	-4.554	0.019
RIMS1	-3.570	0.031
NUDT11	-2.760	0.033
AC051619.7	3.375	0.034
IGHV1-69-2	6.504	0.034
LGR5	-3.760	0.036
RNA5SP150	-12.180	0.036
KCNA1	-3.210	0.036
RP11-254F19.2	-3.303	0.040
BAAT	-4.743	0.042
ACSS3	-2.967	0.046
SPHKAP	-4.804	0.046
LRRC17	-3.132	0.048
**DE cutaneous melanoma**		
**Gene**	**log2FoldChange**	**Adj p value**
IGHV3-21	8.285	4.51×10^{-7}
IGKV4-1	6.313	2.02×10^{-5}
IGLV3-21	7.037	4.80×10^{-4}
IGHV4-59	6.858	7.29×10^{-7}

IGLV6-57	7.853	7.43 x 10^{-4}
IGLV3-1	6.463	7.92 x 10^{-4}
IGKV3-11	6.644	9.08 x 10^{-4}
FDCSP	7.481	1.02 x 10^{-3}
IGHV1-18	5.599	1.02 x 10^{-3}
IGHV4-39	5.939	1.42 x 10^{-3}
IGKV3-15	6.374	1.42 x 10^{-3}
IGHV4-31	5.713	3.18 x 10^{-3}
IGLV1-44	6.218	8.27 x 10^{-3}
TNFRSF11B	3.058	9.56 x 10^{-3}
IGKV3-20	6.053	1.17 x 10^{-2}
IGHGP	6.498	2.13 x 10^{-2}
IGLV2-14	5.584	2.93 x 10^{-2}
IGHG2	5.551	2.93 x 10^{-2}
IGHG1	5.414	2.93 x 10^{-2}
IGKV1-12	8.047	2.93 x 10^{-2}
IGHG4	5.714	3.48 x 10^{-2}
IGKV1-5	5.210	3.48 x 10^{-2}
IGLV2-23	5.790	5.76 x 10^{-2}
IGLV3-25	6.191	5.98 x 10^{-2}
AL445647.1	-8.312	7.49 x 10^{-2}
IGKV3D-20	6.489	7.49 x 10^{-2}
IGHG3	6.304	7.49 x 10^{-2}
IGHV1-46	5.474	7.92 x 10^{-2}
IGKV1-9	6.309	7.92 x 10^{-2}
IGHV5-10-1	8.249	7.92 x 10^{-2}
IGHV1-69D	5.870	8.11 x 10^{-2}
IGHV1-69	8.232	8.49 x 10^{-2}
IGHV3-30	5.431	8.77 x 10^{-2}
IGHV1-24	7.390	8.88 x 10^{-2}
IGHV3-15	5.717	8.91 x 10^{-2}
PRDM7	7.640	0.0001
IGLC3	5.982	0.00012

IGKV3D-11	7.179	0.00013
IGHV3-73	7.382	0.00014
IGHV3-23	5.159	0.00014
LRFN5	-3.622	0.00016
IGHV3-74	5.672	0.00018
IGHA1	5.293	0.0002
IGHV3-33	5.458	0.00023
IGLV1-40	5.986	0.00024
IGHV2-70D	8.089	0.00036
IGHV3-13	7.717	0.00038
IGKC	5.157	0.0005
IGHV6-1	6.956	0.00052
FRRS1L	-4.038	0.00058
MMP3	6.696	0.00065
IGLV1-47	4.633	0.00068
IGLV1-51	5.385	0.00071
IGLC2	4.800	0.00071
IGHA2	4.606	0.00071
IGHV3-66	5.899	0.00071
CD177	4.951	0.000711
IGLV7-46	7.388	0.000711
AC051619.7	3.970	0.000711
GRIA1	-5.828	0.00077
IGKV2-30	4.887	0.00078
POU2AF1	4.180	0.00082
IGHV1-3	5.883	0.00082
IGHM	4.791	0.0009
MEST	-4.380	0.00093
AL136298.1	5.099	0.00093
ADAMTS9-AS1	-2.992	0.00122
IGKV1-27	5.745	0.00128
MMP1	5.032	0.00199
IGHV2-5	4.276	0.00212

IGHV3-11	5.302	0.00231
EPHA7	-4.039	0.0027
IGLV3-19	5.014	0.0029
PTN	-4.187	0.0031
IGLV2-11	5.498	0.0034
IGHV3-48	4.687	0.0035
MZB1	4.425	0.0038
IGLV4-60	7.148	0.0038
TNFRSF12A	1.780	0.0040
ACOXL	3.702	0.0046
IGHV1-69-2	7.427	0.0050
IGHJ4	4.346	0.0050
IGKV1-6	4.164	0.0051
MKRN9P	-5.762	0.0051
AC233755.1	5.532	0.0062
IGHV4-28	5.779	0.0064
IGLV7-43	7.648	0.0066
JCHAIN	3.672	0.0070
SPHKAP	-5.047	0.0079
CLUL1	-4.313	0.008
B4GALNT2	-4.595	0.009
LDB2	-1.852	0.0096
IGHV2-70	4.476	0.0104
MTMR7	-4.773	0.0105
FZD1	-1.952	0.0105
IGLV2-8	4.497	0.0105
AC007938.3	-4.319	0.0108
KCNA1	-3.562	0.0108
FCRL5	3.786	0.0108
ADCY8	-5.651	0.0112
CACNA1G	-4.043	0.0123
LINC01579	-4.998	0.0123
TNFRSF17	-3.279	0.0123

SLC8A3	4.589	0.0123
NUGGC	3.417	0.0126
HPN-AS1	5.849	0.0131
AC087762.2	-6.978	0.0132
LINC02302	-3.582	0.0150
IGLL5	4.165	0.0161
IGHJ3P	4.167	0.0166
CD19	3.982	0.0170
IGHV4-61	5.857	0.0188
SYPL2	-2.216	0.0197
S1PR4	2.650	0.0199
LGR5	-3.959	0.0208
VIPR2	-4.692	0.0217
IGHV3-53	4.610	0.0218
OPRD1	4.253	0.0232
CCL21	3.588	0.0251
AMZ1	3.278	0.0251
CLEC4E	2.179	0.0269
AC084082.1	2.948	0.0270
WNT2	2.481	0.0271
FAM30A	3.557	0.0298
CEMIP	2.141	0.0303
DOK6	-2.258	0.0312
HKDC1	3.708	0.0319
CCN3	3.747	0.0327
IGHV3-20	5.455	0.0327
RN7SL471P	3.365	0.0336
CD22	3.016	0.0343
SNHG18	-2.635	0.0347
U62631.1	3.075	0.0366
FUT9	-5.691	0.0374
UPP1	-1.763	0.0374
MIR4697HG	1.600	0.0374

LINC01013	-3.039	0.0382
OR7E7P	-1.533	0.0386
IGHD	4.341	0.0427
BLK	3.167	0.0448

Table S4. Gene Ontology enrichment in good responder patients with metastatic cutaneous melanoma.

**GO biological process**	**Count**	**Adj p value**
complement activation, classical pathway	61	3.21×10^{-88}
regulation of complement activation	41	1.33×10^{-56}
B cell receptor signaling pathway	41	3.6×10^{-55}
phagocytosis, recognition	39	3.84×10^{-54}
Fc-gamma receptor signaling pathway involved in phagocytosis	40	1.64×10^{-52}
phagocytosis, engulfment	39	1.25×10^{-51}
positive regulation of B cell activation	39	1.05×10^{-47}
Fc-epsilon receptor signaling pathway	37	7.9×10^{-44}
receptor-mediated endocytosis	41	5.28×10^{-43}
leukocyte migration	44	5.81×10^{-43}
immunoglobulin production	28	4.07×10^{-31}
innate immune response	43	1.99×10^{-29}
organelle organization	2	0.00018
glomerular filtration	4	0.000181
antibacterial humoral response	5	0.000623
positive regulation of respiratory burst	3	0.00366
regulation of gene expression	8	0.00696
nucleic acid metabolic process	1	0.00782
regulation of protein oligomerization	4	0.0211
gene expression	1	0.0393
regulation of RNA metabolic process	7	0.0452
protein transport	0	0.0477
**GO molecular function**	**Count**	**Adj p value**
antigen binding	68	7.9×10^{-95}
immunoglobulin receptor binding	40	1.25×10^{-56}
signalling receptor binding	51	2×10^{-22}
binding	106	7.85×10^{-5}
organic cyclic compound binding	10	0.000131
heterocyclic compound binding	10	0.000175
nucleic acid binding	4	0.000452
metal ion binding	6	0.00487
cation binding	8	0.0438
**GO cellular component**	**Count**	**Adj p value**
external side of plasma membrane	41	1.36×10^{-36}
blood microparticle	21	5.75×10^{-21}
membrane-enclosed lumen	5	5.26×10^{-7}
intracellular organelle lumen	5	5.49×10^{-7}
organelle lumen	5	5.74×10^{-7}
monomeric IgA immunoglobulin complex	4	4.04×10^{-6}
cytosol	6	7.65×10^{-6}
nuclear lumen	3	8.82×10^{-6}
nucleoplasm	2	2.55×10^{-5}
secretory dimeric IgA immunoglobulin complex	3	0.00014

pentameric IgM immunoglobulin complex	3	0.000151
integral component of postsynaptic density membrane	4	0.0103
extracellular exosome	24	0.0156
cytoskeletal part	1	0.0471

Table S5. Person correlation between TPM values from RNA-seq and △Ct values from TaqMan.

Gene	Pearson correlation	P-value
TNFRSF11B	0.80795	0.05179
IGLV6-57	0.81485	0.09293
IGHA1	0.94815	0.00397
GRIA1	0.99773	0.00013
Overall	0.78756	0.00001

Table S6. List of the genes of the transcriptomic response signature of the metastatic cutaneous melanoma cohort associated with PFS and OS

Gene	Logrank OS	HR OS	Logrank PFS	HR PFS	B cell related-gene
IGHV3-21	3.00×10^{-4}	Ref low	0.0025	Ref low	Yes
		med: 0 (CI: 0-Inf)		med: 0.0907 (CI: 0.0119-0.6928)	
		high: 0.0976 (CI: 0.018-0.5309)		high: 0.0907 (CI: 0.0159-0.5175)	
IGKV4-1	0	Ref low	5.00×10^{-4}	Ref low	Yes
		med: 1.0241 x 109 (CI: 0-Inf)		med: 3.8757 (CI: 0.3557-42.2305)	
		high: 0.2009 (CI: 0.0182-2.2195)		high: 0.2031 (CI: 0.0184-2.2428)	
IGLV3-21	0.0079	Ref low	0.0179	Ref low	Yes
		med: 0.091 (CI: 0.0131-0.633)		med: 0.1445 (CI: 0.0253-0.8248)	
		high: 0.1484 (CI: 0.0296-0.7435)		high: 0.1513 (CI: 0.0299-0.7664)	
IGHV4-59	0.0251	Ref low	0.2206	Ref low	Yes
		med: 0.117 (CI: 0.0184-0.7427)		med: 0.5048 (CI: 0.1324-1.9239)	
		high: 0.2199 (CI: 0.0453-1.0672)		high: 0.2953 (CI: 0.0684-1.2752)	
IGLV6-57	2.00×10^{-4}	Ref low	4.00×10^{-4}	Ref low	Yes
		med: 0.0267 (CI: 0.0024-0.2924)		med: 0.0505 (CI: 0.0055-0.461)	
		high: 0.0539 (CI: 0.0053-0.5456)		high: 0.0323 (CI: 0.0027-0.3925)	
IGLV3-1	0.0078	Ref low	0.0019	Ref low	Yes

		med: 0.1632 (CI: 0.0289-0.9222)		med: 0.3252 (CI: 0.0691-1.5314)	
		high: 0.1043 (CI: 0.0192-0.5671)		high: 0.0464 (CI: 0.0061-0.3525)	
FDCSP	0.0752	Ref low	0.0344	Ref low	Yes
		med: 0.2611 (CI: 0.0309-2.2089)		med: 1.0625 (CI: 0.2011-5.6146)	
		high: 0.2605 (CI: 0.07-0.969)		high: 0.2094 (CI: 0.0559-0.7837)	
IGKV3-15	0.0082	Ref low	0.0151	Ref low	Yes
		med: 0.1051 (CI: 0.0184-0.6)		med: 0.1792 (CI: 0.0367-0.876)	
		high: 0.1508 (CI: 0.0278-0.8169)		high: 0.1097 (CI: 0.0182-0.6631)	
IGLV1-44	0.0042	Ref low	0.0135	Ref low	No
		med: 0.0444 (CI: 0.004-0.4925)		med: 0.164 (CI: 0.0323-0.8325)	
		high: 0.1615 (CI: 0.0295-0.8832)		high: 0.1396 (CI: 0.0267-0.7298)	
TNFRSF11B	0.0261	Ref low	0.04	Ref low	Yes
		med: 0.2121 (CI: 0.0443-1.0154)		med: 0.207 (CI: 0.0438-0.9782)	
		high: 0.0655 (CI: 0.0057-0.755)		high: 0.1178 (CI: 0.0159-0.8733)	
IGKV3-20	0.0441	Ref low	0.1061	Ref low	Yes
		med: 0.3113 (CI: 0.0442-2.192)		med: 0.3549 (CI: 0.0507-2.4867)	
		high: 0.1679 (CI: 0.0352-0.7998)		high: 0.2156 (CI: 0.0459-1.0124)	
IGHGP	3.00×10^{-4}	Ref low	8.00×10^{-4}	Ref low	Yes

		med: 5.6525 (CI: 0.4817-66.3249)		med: 6.57 (CI: 0.5474-78.8574)	
		high: 0.1117 (CI: 0.0201-0.6222)		high: 0.1832 (CI: 0.0398-0.844)	
IGKV1-12	0.0076	Ref low	0.1134	Ref low	Yes
		med: 0.0516 (CI: 0.0048-0.5529)		med: 0.2809 (CI: 0.0701-1.1265)	
		high: 0.1194 (CI: 0.0121-1.1808)		high: 0.3339 (CI: 0.0668-1.6696)	
IGHG4	0.017	Ref low	0.0377	Ref low	Yes
		med: 1.3105 (CI: 0.2095-8.1953)		med: 1.4902 (CI: 0.2295-9.6758)	
		high: 0.186 (CI: 0.0295-1.1712)		high: 0.271 (CI: 0.0462-1.589)	
IGKV1-5	0.0192	Ref low	0.0442	Ref low	Yes
		med: 1.0869 (CI: 0.175-6.7529)		med: 0.7264 (CI: 0.1149-4.5917)	
		high: 0.167 (CI: 0.0265-1.054)		high: 0.1735 (CI: 0.0291-1.0364)	
IGLV2-23	0.0084	Ref low	0.0169	Ref low	Yes
		med: 0.161 (CI: 0.0248-1.0446)		med: 0.1791 (CI: 0.031-1.0358)	
		high: 0.1103 (CI: 0.0206-0.5915)		high: 0.1341 (CI: 0.0267-0.6733)	
IGLV3-25	0.03	Ref low	0.0992	Ref low	Yes
		med: 0.168 (CI: 0.0275-1.0266)		med: 0.7662 (CI: 0.1982-2.9619)	
		high: 0.17 (CI: 0.0341-0.8481)		high: 0.2289 (CI: 0.0529-0.9913)	
IGHG3	4.00×10^{-4}	Ref low	0	Ref low	Yes

		med: 6.8185 (CI: 0.5044-92.1811)		med: 6.8692 x 10^8 (CI: 0-Inf)	
		high: 0.1903 (CI: 0.0312-1.1593)		high: 0.2594 (CI: 0.0428-1.5702)	
IGHV1-46	0.0089	Ref low	0.0176	Ref low	Yes
		med: 0.1228 (CI: 0.0246-0.6133)		med: 0.1542 (CI: 0.0325-0.7323)	
		high: 0.1335 (CI: 0.0191-0.9324)		high: 0.1314 (CI: 0.0184-0.938)	
IGKV1-9	0.4467	Ref low	0.0271	Ref low	Yes
		med: 0.425 (CI: 0.0937-1.9271)		med: 0.2044 (CI: 0.0424-0.9858)	
		high: 0.4843 (CI: 0.1183-1.9832)		high: 0.1406 (CI: 0.0254-0.7797)	
IGHV1-69D	0.0163	Ref low	0.0446	Ref low	Yes
		med: 0.1092 (CI: 0.0174-0.6836)		med: 0.2567 (CI: 0.0589-1.1177)	
		high: 0.2089 (CI: 0.0433-1.0076)		high: 0.1727 (CI: 0.0344-0.8678)	
IGHV1-69	0.0091	Ref low	0.0668	Ref low	No
		med: 0.0696 (CI: 0.0071-0.682)		med: 0.4625 (CI: 0.132-1.6209)	
		high: 0.0832 (CI: 0.0076-0.9065)		high: 0.1189 (CI: 0.014-1.011)	
IGHV3-15	0.0082	Ref low	0.0151	Ref low	Yes
		med: 0.1051 (CI: 0.0184-0.6)		med: 0.1792 (CI: 0.0367-0.876)	
		high: 0.1508 (CI: 0.0278-0.8169)		high: 0.1097 (CI: 0.0182-0.6631)	
LRFN5	6.00 x 10^{-4}	Ref low	0.0019	Ref low	Yes

		med: 0.0454 (CI: 0.0024-0.8437)		med: 0.0542 (CI: 0.0038-0.7703)	
		high: 0.9946 (CI: 0.1073-9.2177)		high: 0.5815 (CI: 0.0589-5.742)	
IGHV3-74	0.0185	Ref low	0.0201	Ref low	No
		med: 0.1854 (CI: 0.0372-0.9241)		med: 0.338 (CI: 0.0844-1.3537)	
		high: 0.1319 (CI: 0.023-0.755)		high: 0.0915 (CI: 0.0139-0.6032)	
IGHA1	2.00×10^{-4}	Ref low	3.00×10^{-4}	Ref low	Yes
		med: 9.6440×10^{8} (CI: 0-Inf)		med: 4.9093×10^{8} (CI: 0-Inf)	
		high: 0.262 (CI: 0.0474-1.4495)		high: 0.3402 (CI: 0.0618-1.8736)	
IGLV1-40	0.3826	Ref low	0.0167	Ref low	Yes
		med: 0.6225 (CI: 0.1349-2.8728)		med: 0.2755 (CI: 0.0582-1.3038)	
		high: 0.3789 (CI: 0.0924-1.5542)		high: 0.1045 (CI: 0.0182-0.5998)	
FRRS1L	2.00×10^{-4}	Ref low	0	Ref low	No
		med: 41.3199 (CI: 3.132-545.1214)		med: 5.8939×10^{9} (CI: 0-Inf)	
		high: 9.2464 (CI: 0.5781-147.8784)		high: 9.4868 (CI: 0.5928-151.8167)	
IGLV1-47	0.0072	Ref low	0.0173	Ref low	Yes
		med: 0.0947 (CI: 0.0164-0.5481)		med: 0.1612 (CI: 0.0327-0.7952)	
		high: 0.1689 (CI: 0.0316-0.9026)		high: 0.129 (CI: 0.0216-0.7726)	
IGLV1-51	0.0118	Ref low	0.0206	Ref low	Yes

		med: 0.098 (CI: 0.0144-0.6673)		med: 0.1802 (CI: 0.034-0.9555)	
		high: 0.1132 (CI: 0.0159-0.8064)		high: 0.0988 (CI: 0.0147-0.6654)	
AC051619.8	0.0188	Ref low	0.0305	Ref low	Yes
		med: 0.404 (CI: 0.1045-1.561)		med: 0.5936 (CI: 0.1756-2.0062)	
		high: 0.065 (CI: 0.0067-0.6305)		high: 0.0861 (CI: 0.01-0.7419)	
IGKV2-30	0.0076	Ref low	0.113	Ref low	No
		med: 0.0599 (CI: 0.0064-0.5624)		med: 0.2865 (CI: 0.0809-1.0148)	
		high: 0.1744 (CI: 0.0118-2.5842)		high: 0.3726 (CI: 0.0439-3.1621)	No
IGHV1-3	0.0088	Ref low	0.0097	Ref low	
		med: 0.1298 (CI: 0.0254-0.6627)		med: 0.1958 (CI: 0.0419-0.9151)	
		high: 0.1187 (CI: 0.0191-0.736)		high: 0.0715 (CI: 0.0096-0.5328)	Yes
IGHM	0.0482	Ref low	0.1216	Ref low	
		med: 0.3421 (CI: 0.0338-3.4658)		med: 0.1878 (CI: 0.017-2.08)	
		high: 0.1695 (CI: 0.035-0.8213)		high: 0.2433 (CI: 0.0532-1.1137)	Yes
AL136298.1	0.0213	Ref low	0.0195	Ref low	
		med: 0.1921 (CI: 0.0403-0.9147)		med: 0.2301 (CI: 0.0607-0.873)	
ADAMTS9-AS1	9.00×10^{-4}	Ref low	0.0058	Ref low	Yes
		med: 9.0733 (CI: 1.6194-50.837)		med: 8.1941 (CI: 1.4537-46.1881)	

		high: 42.5152 (CI: 2.0044-901.7862)		high: 18.8478 (CI: 1.2622-281.4478)	
IGKV1-27	2.00 x 10^{-4}	Ref low	4.00 x 10^{-4}	Ref low	No
		med: 0.0395 (CI: 0.0044-0.3566)		med: 0.0488 (CI: 0.0056-0.4289)	
		high: 0.0281 (CI: 0.0016-0.4909)		high: 0.0244 (CI: 0.0014-0.4352)	
IGHV2-5	0.0305	Ref low	0.0185	Ref low	No
		med: 0.2043 (CI: 0.0401-1.0405)		med: 0.1754 (CI: 0.0348-0.8839)	
		high: 0.1358 (CI: 0.0241-0.7638)		high: 0.1265 (CI: 0.023-0.695)	
IGHV3-11	0.0196	Ref low	0.046	Ref low	Yes
		med: 0.158 (CI: 0.0333-0.7508)		med: 0.2383 (CI: 0.0583-0.9746)	
		high: 0.1603 (CI: 0.0249-1.0327)		high: 0.1678 (CI: 0.0275-1.0248)	
EPHA7	0.0128	Ref low	0.0278	Ref low	Yes
		med: 2.1752 (CI: 0.5307-8.9153)		med: 1.6648 (CI: 0.4342-6.3833)	
		high: 9.5815 (CI: 1.7125-53.6074)		high: 7.4869 (CI: 1.4033-39.9447)	
PTN	0.0034	Ref low	0.0044	Ref low	No
		med: 0.1001 (CI: 0.008-1.2542)		med: 0.0868 (CI: 0.0072-1.05)	
		high: 1.0269 (CI: 0.1086-9.7083)		high: 0.6459 (CI: 0.0636-6.5572)	
IGLV2-11	0.0064	Ref low	0.0078	Ref low	No
		med: 0.108 (CI: 0.0162-0.7187)		med: 0.2359 (CI: 0.0528-1.0528)	

		high: 0.1044 (CI: 0.0162-0.6724)		high: 0.0737 (CI: 0.0107-0.5089)	
IGHV3-48	0.0082	Ref low	0.0151	Ref low	No
		med: 0.1051 (CI: 0.0184-0.6)		med: 0.1792 (CI: 0.0367-0.876)	
		high: 0.1508 (CI: 0.0278-0.8169)		high: 0.1097 (CI: 0.0182-0.6631)	
SPHKAP	0.0325	Ref low	0.0906	Ref low	No
		med: 5.9837 (CI: 1.3146-27.237)		med: 4.3077 (CI: 1.0158-18.2671)	
		high: 3.0111 (CI: 0.3286-27.5901)		high: 2.7262 (CI: 0.3056-24.3194)	
B4GALNT2	0	Ref low	0	Ref low	No
		med: 2.6671 x 10^{9} (CI: 0-Inf)		med: 4.2404 x 10^{18} (CI: 0-Inf)	
		high: 37718 x 10^{9} (CI: 0-Inf)		high: 1.4774 x 10^{9} (CI: 0-Inf)	
MTMR7	9.00 x 10^{-4}	Ref low	0.0058	Ref low	No
		med: 9.0733 (CI: 1.6194-50.837)		med: 8.1941 (CI: 1.4537-46.1881)	
		high: 42.5152 (CI: 2.0044-901.7862)		high: 18.8478 (CI: 1.2622-281.4478)	
AC007938.3	0.051	Ref low	0.0344	Ref low	No
		med: 0.9315 (CI: 0.1797-4.8296)		med: 0.3664 (CI: 0.0451-2.9784)	
		high: 5.4337 (CI: 1.1441-25.8071)		high: 4.8387 (CI: 1.0384-22.5464)	
KC1	0	Ref low	0	Ref low	No
		med: 8.144 (CI: 1.5969-41.5347)		med: 8.144 (CI: 1.5969-41.5347)	

		high: 7735124113.7234 (CI: 0-Inf)		high: 7735124047.9921 (CI: 0-Inf)	No
ADCY8	1.00 x 10^{-4}	Ref low	0.0017	Ref low	
		med: 0.9686 (CI: 0.1993-4.707)		med: 0.8569 (CI: 0.1805-4.0681)	
		high: 2244862651.0162 (CI: 0-Inf)		high: 21.3621 (CI: 1.8564-245.8251)	No
CAC1G	0.0051	Ref low	0.026	Ref low	
		med: 1.4203 (CI: 0.2705-7.4573)		med: 1.0259 (CI: 0.2428-4.3347)	
		high: 10.9107 (CI: 1.5374-77.4311)		high: 6.3787 (CI: 1.058-38.4582)	No
NUGGC	0.174	Ref low	0.0379	Ref low	
		med: 0.4773 (CI: 0.1047-2.1754)		med: 1.3702 (CI: 0.3687-5.0919)	
		high: 0.2089 (CI: 0.0371-1.1753)		high: 0.1917 (CI: 0.0339-1.0841)	No
HPN-AS1	0.6337	Ref low	0.0481	Ref low	
		med: 1.0894 (CI: 0.241-4.924)		med: 0.6679 (CI: 0.1574-2.8339)	
		high: 0.5719 (CI: 0.1415-2.311)		high: 0.1839 (CI: 0.0421-0.8037)	No
AC087762.2	0.0253	Ref low	0.1386	Ref low	
		med: 2.5884 (CI: 0.6896-9.7156)		med: 1.2293 (CI: 0.3451-4.3788)	Yes
		high: 11.7773 (CI: 1.4101-98.3621)		high: 5.1146 (CI: 0.8628-30.3179)	
LINC02302	0.0515	Ref low	0.0142	Ref low	No

		med: 1.579 (CI: 0.4211-5.9204)		med: 4.793 (CI: 1.1473-20.0242)	
		high: 9.1712 (CI: 1.1495-73.1698)		high: 11.6396 (CI: 1.5512-87.3381)	
SYPL2	0.0069	Ref med	0.0024	Ref med	No
		high: 6.271 (CI: 1.3764-28.5708)		high: 7.5818 (CI: 1.6504-34.8297)	
S1PR4	0.0114	Ref low	0.0256	Ref low	
		med: 0.1448 (CI: 0.0302-0.6943)		med: 0.1901 (CI: 0.0411-0.8801)	No
		high: 0.0767 (CI: 0.0066-0.8868)		high: 0.0757 (CI: 0.0065-0.8818)	
LGR5	0	Ref low	0	Ref low	
		med: 0.5269 (CI: 0.1045-2.657)		med: 0.6149 (CI: 0.1531-2.4696)	No
		high: 2361281642.1492 (CI: 0-Inf)		high: 2.4803 x 10^9 (CI: 0-Inf)	
AMZ1	0.0267	Ref low	0.0301	Ref low	
		med: 0.1504 (CI: 0.0234-0.9656)		med: 0.1933 (CI: 0.0367-1.0176)	No
		high: 0.0817 (CI: 0.0089-0.746)		high: 0.0948 (CI: 0.0122-0.7365)	
AC084082.1	0.0011	Ref low	0.002	Ref low	
		med: 2.5332 (CI: 0.4184-15.337)		med: 3.6881 (CI: 0.6344-21.4393)	No
		high: 0.096 (CI: 0.0087-1.0632)		high: 0.3249 (CI: 0.054-1.9541)	
CEMIP	0.0646	Ref med	0.0122	Ref med	

		high: 0.1432 (CI: 0.013-1.5805)		high: 0.069 (CI: 0.0043-1.1037)	Yes
DOK6	0.0412	Ref med	0.058	Ref med	
		high: 3.4016 (CI: 0.988-11.7111)		high: 3.0235 (CI: 0.9205-9.9315)	
CD22	0.0154	Ref med	0.0356	Ref med	Yes
		high: 0.2081 (CI: 0.0514-0.8431)		high: 0.2649 (CI: 0.0702-1.0001)	
SNHG18	0.0312	Ref low	0.0529	Ref low	
		med: 0.1685 (CI: 0.0158-1.7974)		med: 0.1265 (CI: 0.0111-1.4407)	No
		high: 1.0678 (CI: 0.1041-10.9579)		high: 0.607 (CI: 0.0561-6.5726)	
U62631.1	0.0402	Ref low	0.0406	Ref low	
		med: 0.2167 (CI: 0.0492-0.9554)		med: 0.3405 (CI: 0.0831-1.3947)	No
		high: 0.1297 (CI: 0.0182-0.9247)		high: 0.0678 (CI: 0.0062-0.7429)	
FUT9	1.00×10^{-4}	Ref low	0.0013	Ref low	
		med: 2.7133 (CI: 0.3024-24.3479)		med: 2.6985 (CI: 0.3008-24.2078)	No
		high: 2385660655.1196 (CI: 0-Inf)		high: 24.8065 (CI: 2.1301-288.8926)	
LINC01013	0.0065	Ref low	0.0794	Ref low	
		med: 1.320×10^{8} (CI: 0-Inf)		med: 0.494 (CI: 0.0575-4.2452)	No
		high: 1.3594×10^{9} (CI: 0-Inf)		high: 2.8981 (CI: 0.2388-35.1671)	
OR7E7P	0	Ref low	5.00×10^{-4}	Ref low	

		med: 1.3352 x 10^8 (CI: 0-Inf)		med: 1.1713 x 10^8 (CI: 0-Inf)	No
		high: 3.7417 x 10^{18} (CI: 0-Inf)		high: 1.5156 x 10^9 (CI: 0-Inf)	

Table S7. Association of the stromal immune cells to response to anti-PD1 treatment in the single-cell RNA-seq validation cohort and bulk deconvolution.

Stromal immune cell type in scRNA-seq	Wilcoxon test	Response association
B cells	0.04069	Good
CD4 cytotoxic	0.17695	-
CD4 self renewing	0.32569	-
CD8 T cytotoxic 1	0.41401	-
CD8 T cytotoxic 2	0.41277	-
CD8 T exhausted	0.32612	-
CD8 T memory	0.93455	-
DC plasmacytoid	0.01027	Bad
NK cells	0.56745	-
Plasma cells	0.46202	-
Macrophages	0.02939	Bad
T gamma delta	0.01556	Bad
T reg	0.43713	-
**B cell type-related in scRNA-seq**	**Wilcoxon test**	
Naive B cells	0.040428	Good
Naive B cells IGK^{+}	0.03202	Good
Naive B cells IGL^{+}	0.05769	-
Plasmablasts	0.72838	-
Plasma cells	0.46202	-
**Estimation of cell type - MCPcounter**	**Wilcoxon test**	**Response association**
T cells	0.25227	-
CD8 T cells	0.46975	-
Cytotoxic lymphocytes	0.68059	-
B lineage	0.04173	Good
NK cells	0.21048	-
Monocytic lineage	0.21048	-
Myeloid dendritic cells	0.40786	-
Neutrophils	0.91818	-
Endothelial cells	0.91818	-
Fibroblasts	1	-
**Estimation of cell type - CIBERSORTx**	**Wilcoxon test**	**Response association**
CD4 cytotoxic	0.45538	-

CD4 self renewing	0.23165	-
CD8 T cytotoxic 1	0.120478	-
CD8 T cytotoxic 2	1	-
CD8 T exhausted	0.68059	-
CD8 T memory	0.11778	-
DC plasmacytoid	0.62646	-
NK cells	0.44969	-
Macrophages	0.29912	-
T gamma delta	0.75769	-
T reg	0.29912	-
Naive B cells	0.07110	-
Naive B cells IGK$^+$	0.53452	-
Naive B cells IGL$^+$	0.13807	-
Plasmablasts	0.12047	-
Plasma cells	0.04178	Good

Table S8. Common genes DE in good responders in the bulk RNA-seq and the scRNA-seq cohorts of metastatic melanoma patients treated with anti-PD1.

Genes	Bulk RNA-seq adj p-value	scRNA-seq adj p-value
CD19	8.503637×10^{-5}	7.609444×10^{-50}
IGHM	2.507395×10^{-6}	2.022739×10^{-36}
CD22	2.082801×10^{-4}	7.161602×10^{-33}
IGHG3	7.897861×10^{-8}	1.671405×10^{-32}
IGHGP	1.405069×10^{-8}	4.514980×10^{-29}
IGHG2	2.309965×10^{-8}	2.297382×10^{-25}
POU2AF1	2.201943×10^{-6}	2.910002×10^{-10}
UPP1	2.373111×10^{-4}	1.136566×10^{-8}

Table S9. Spearman correlation between events values of the Multispectral Immunoflourescence validation and the deconvolution proportion inferred from RNA-seq.

Immunofluorescence	Deconvolution	Spearman correlation	P-value
Plasma cell-like	Plasma cells	0.80511	0.01590
CD19	Naive B cells IGL$^+$	0.87426	0.00451
CD19	Naive B cells IGK$^+$	0.89821	0.00243
CD19	Plasma cells	0.72891	0.04022
CD20	Naive B cells	0.79090	0.03418

Table S10. Ranking of counts of the 100 clonotypes that are enriched in good responders to Nivolumab.

Sample ID	Response	Clonotypes counts
IMK26	Good	11317
IMK31	Good	9169
IMK38	Good	3971
IMK30	Good	1845
IMK36	Bad	1732
IMK27	Good	561
IMK24	Good	233
IMK32	Good	164
IMK48	Good	146
IMK22	Bad	126
IMK23	Good	78
IMK35	Bad	40
IMK34	Bad	39
IMK37	Bad-severe	16
IMK39	Bad-severe	8
IMK21	Bad-severe	0

Table S11. Predictive evaluation of models in external cohorts.

Cohort	# Good responders	# Bad responders	Treatment	TIDE	IMK-140	IMK-RF
Gide et al., Cancer Cell 2019 (PD1+antiCTLA4)	21	11	Anti-PD1+anti-CTLA4	0.62	0.66	0.69
Gide et al., Cancer Cell 2019 (PD1)	19	22	Anti-PD1	0.60	0.72	0.81
Hugo et al., Cell 2016	14	12	Anti-PD1	0.84	0.7	0.62
Nathanson et al., Cancer Immunol Res 2017	8	16	Anti-CTLA4	0.45	0.52	0.8
Riaz et al., Cell 2017 (naive)	6	19	Anti-PD1	0.45	0.46	0.61
Riaz et al., Cell 2017 (prior CTLA4)	4	22	Anti-PD1	0.23	0.71	0.84
Liu et al., Nat Medicine 2019 (naive)	27	47	Anti-PD1	0.4	0.55	0.64
Liu et al., Nat Medicine 2019 (prior CTLA4)	17	30	Anti-PD1	0.4	0.64	0.52

Table S12. Clinicopathological characteristics of the Discovery cohort.

Parameter	Value	Good responders	Bad responders	Lost values
**Age at start IT**	56.39 [19.1 - 76.5]	62.41 [19.1 - 74.38]	56.39 [36.65 - 76.5]	1 (5%)
**Sex**				0 (0%)
Female	10 (47.62 %)	6 (54.55 %)	4 (40 %)	
Male	11 (52.38 %)	5 (45.45 %)	6 (60 %)	
**Stage at diagnosis**				3 (0.14%)
Stage I	2 (11.11 %)	1 (12.5 %)	1 (10 %)	
Stage II	6 (33.33 %)	3 (37.5 %)	3 (30 %)	
Stage III	4 (22.22 %)	4 (50 %)		
Stage IV	6 (33.33 %)		6 (60 %)	
**Diagnosis subtype**				0 (0%)
Cutaneous	16 (76.19 %)	9 (81.82 %)	7 (70 %)	
Mucosal	2 (9.52 %)		2 (20 %)	
Uveal	3 (14.29 %)	2 (18.18 %)	1 (10 %)	
**Diabetes**				1 (0.05%)
No	18 (90 %)	9 (90 %)	9 (90 %)	
Yes	2 (10 %)	1 (10 %)	1 (10 %)	
**Dyslipidemia**				1 (0.05%)
No	17 (85 %)	7 (70 %)	10 (100 %)	
Yes	3 (15 %)	3 (30 %)		
**Hypertension**				1 (0.05%)
No	9 (45 %)	3 (30 %)	6 (60 %)	
Yes	11 (55 %)	7 (70 %)	4 (40 %)	
**BRAF**				1 (0.05%)
Wild-type	15 (75 %)	9 (90 %)	6 (60 %)	
Mutated	5 (25 %)	1 (10 %)	4 (40 %)	
**Lung metastasis**				0 (0%)
No	8 (38.1 %)	1 (9.09 %)	7 (70 %)	
Yes	13 (61.9 %)	10 (90.91 %)	3 (30 %)	
Lymph node metastasis				0 (0%)
No	9 (42.9 %)	6 (54.5 %)	3 (30 %)	
Yes	12 (57.1 %)	5 (45.5 %)	7 (70 %)	
**CNS metastasis**				0 (0%)
No	9 (42.86 %)	6 (54.55 %)	3 (30 %)	
Yes	12 (57.14 %)	5 (45.45 %)	7 (70 %)	
**No. metastasis**	2 [1 - 4]	2.5 [1 - 4]	2 [1 - 4]	4 (0.19%)

**LDH first metastasis (U/L)**	167 [107 - 498]	215 [164 - 498]	148.5 [107 - 330]	11 (0.52%)
**LDH previous to IT (U/L)**	254.5 [107 - 801]	260 [208 - 498]	249 [107 - 801]	9 (0.43%)
**Lymphocytes previous to IT (counts x 10^6/L)**	1000 [370 - 2300]	1580 [370 - 2300]	900 [500 - 1520]	5 (0.24%)
**Neutrophiles previous to IT (counts x 10^6/L)**	5635 [2650 - 18800]	4300 [2650 - 11600]	6800 [3490 - 18800]	3 (0.14%)
**Platelets previous to IT (counts x 10^6/L)**	269500 [148000 - 544000]	233000 [159000 - 330000]	281000 [148000 - 544000]	3 (0.14%)
**No. Previous lines**	0 [0 - 3]	0 [0 - 2]	1 [0 - 3]	4 (0.19%)
**Toxicity IT**				0 (0%)
No	7 (33.33 %)	1 (9.09 %)	6 (60 %)	
Yes	14 (66.67 %)	10 (90.91 %)	4 (40 %)	
**Maximum toxicity grade**	2 [1 - 3]	2 [1 - 3]	2 [2 - 3]	10 (0.48%)

L=Litres; U/L=Units per litre
